# Pioneering predictions of AKI and AKIN severity in burn patients: a comprehensive CBC approach

**DOI:** 10.1038/s41598-024-51253-x

**Published:** 2024-01-05

**Authors:** Jongsoo Park, Dohern Kym, Myongjin Kim, Yong Suk Cho, Jun Hur, Wook Chun, Dogeon Yoon, Jaechul Yoon

**Affiliations:** 1 Department of Surgery and Critical Care, Burn Center, Hangang Sacred Heart Hospital, Hallym University Medical Center, 12, Beodeunaru-ro 7-gil, Youngdeungpo-gu, Seoul, 07247 South Korea; 2 Burn Institutes, Hangang Sacred Heart Hospital, Hallym University Medical Center, 12, Beodeunaru-ro 7-gil, Youngdeungpo-gu, Seoul, 07247 South Korea

**Keywords:** Biomarkers, Medical research, Risk factors

## Abstract

This study aims to evaluate the utility of complete blood count (CBC) markers, in conjunction with the acute kidney injury network (AKIN) criteria, for the early detection, severity assessment, and prediction of mortality outcomes of acute kidney injury (AKI) in burn patients. The research seeks to fill existing gaps in knowledge and validate the cost-effectiveness of using CBC as a routine diagnostic tool for better management of AKI. The study was conducted at Hangang Sacred Heart Hospital. We performed a large-scale retrospective analysis of 2758 adult patients admitted to the burn intensive care unit over a 12-year period. Among these patients, AKI occurred in 1554 patients (56.3%). Based on the AKIN stage classification, 794 patients (28.8%) were categorized as AKIN 1, 494 patients (17.9%) as AKIN 2, and 266 patients (9.6%) as AKIN 3. We defined several ratio markers, including the Neutrophil-to-lymphocyte ratio (NLR), Platelet-to-lymphocyte ratio (PLR), Monocyte-to-lymphocyte ratio (MLR), systemic immune-inflammation index (SII), and various mean platelet volume (MPV) ratios. Our statistical analyses, conducted using the R programming language, revealed significant correlations between these markers and AKI severity. The AUC values for neutrophil count and WBC count were 0.790 and 0.793, respectively, followed by immature granulocyte count with an AUC of 0.727. For red blood cell (RBC)-related parameters, the AUC values for hematocrit (Hct), hemoglobin (Hb), and RBC count were 0.725, 0.713, and 0.713, respectively. Among the platelet-related parameters, only platelet distribution width (PDW) had an AUC of 0.677. Among the ratio markers, the NLR had the highest AUC at 0.772, followed by MPVNR and SII with AUC values of 0.700 and 0.680, respectively. The findings underscore the potential of CBC as an economical, routine test for AKI, thereby paving the way for enhanced patient outcomes. Our study suggests the utility of routine CBC tests, specifically WBC count and PLR, for predicting AKI and platelet, MPV, and NLR for mortality assessment in burn patients. These findings underscore the potential of easily accessible CBC tests in enhancing AKI management. However, further multicenter studies are needed for validation.

## Introduction

Acute kidney injury (AKI) is a severe and frequent complication in burn patients, often leading to increased morbidity and mortality. The early detection and accurate assessment of AKI severity are pivotal for effective management and improved patient outcomes^1^. However, the early detection of AKI, particularly in burn patients, remains a significant clinical challenge due to the complex interplay of various physiological factors and the often-subtle onset of AKI. This challenge underscores the need for reliable, cost-effective, and easily accessible biomarkers that can aid in the early detection and severity assessment of AKI in burn patients^2,3^.

The acute kidney injury network (AKIN) criteria, which classifies AKI based on changes in serum creatinine or urine output, provides a standardized framework for assessing AKI severity^4^. However, the AKIN criteria alone may not be sufficient for early detection and severity assessment of AKI in burn patients^5^. Therefore, there is a growing interest in exploring additional biomarkers that can complement the AKIN criteria in predicting AKI severity and mortality outcomes in burn patients^6^.

By focusing on CBC—a routine, cost-effective test—we aim to push the envelope in AKI diagnostics. While CBC tests are traditionally used for assessing a patient's general health status and immune response, their application has largely been overlooked in the realm of AKI detection and severity assessment^7^. CBC-related tests, specifically red cell width distribution (RDW), neutrophil to lymphocyte ratio (NLR), neutrophil to platelet ratio (NPR), monocyte to lymphocyte ratio (MLR), platelet to lymphocyte ratio (PLR), mean platelet volume (MPV), platelet count, have been identified as potential indicators of systemic inflammation and stress, which are common in burn patients and can contribute to the development of AKI.

However, the relationship between these CBC markers AKI, particularly in the context of burn patients, remains poorly understood, and only a limited number of studies have reported on this topic. Existing studies provide some insights into the potential of these markers in predicting AKI severity and mortality outcomes, but there are significant gaps and uncertainties that need to be addressed^8^. For instance, the predictive power of these markers may vary depending on the patient’s demographic and clinical characteristics, the severity and extent of the burn injury, and the presence of other comorbidities^9^. Furthermore, the optimal cut-off values for these markers in predicting AKI severity and mortality outcomes in burn patients are yet to be established.

This thesis aims to investigate the diagnostic utility of CBC markers in conjunction with AKIN criteria to predict the presence or absence, severity, and mortality outcomes based on renal function of AKI in burn patients, thereby addressing existing knowledge gaps to enhance patient care and validate the cost-effective use of CBC as a routine diagnostic tool for AKI.

## Method

### Study site and patients

This large-scale retrospective study was conducted at Hallym University Hangang Sacred Heart Hospital, a tertiary care center with a specialized burn intensive care unit. The study adheres to the STROBE guidelines for observational studies. The study population comprised adult patients (18 years and older) admitted to the burn intensive care unit at Hallym University Hangang Sacred Heart Hospital over a 12-year period, from January 2010 to December 2022. Patients were included if they had sustained burn injuries and had been admitted to the burn intensive care unit during the study period. Patients were considered eligible for admission to the Burn Intensive Care Units (BICUs) if they met one or more of the following criteria: total body surface area (TBSA) burns covering 20% or more in the general adult population, or 10% or more in pediatric patients aged 10 years or younger and elderly individuals aged 65 years or older. Additional qualifications for admission included full-thickness burns affecting a minimum of 10% TBSA, incidents involving high-voltage electrical burns, burn injuries concomitant with significant trauma or inhalation injury, and individuals deemed to be at high risk due to specific medical or situational factors. Patients were excluded if they were under 18 years of age, had not sustained burn injuries, and had preexisting renal diseases. The study was conducted in accordance with the principles outlined in the Declaration of Helsinki. Approval was obtained from the Institutional Review Board of Hangang Sacred Heart Hospital (HG 2022-011). Informed consent from participants was waived due to the retrospective nature and design of the study.

### General burn management

The management of burn injuries adhered to the standardized protocols established by the Burn Center at Hangang Sacred Heart Hospital. Fluid resuscitation was administered in accordance with either the Parkland formula^10^ for burns covering less than 40% of total body surface area (TBSA) or the Warden formula^11^ for burns encompassing 40% TBSA or more. In the absence of contraindications such as ileus or ongoing burn shock, enteral nutrition was initiated within 48 h post-injury. Nutritional needs were subsequently reviewed and adjusted during weekly meetings by the nutritional support team. Pain-managed wound dressing was conducted daily. Initial excision of the burn wound and allograft application were generally performed within 5 days post-injury, followed by autografting procedures. Antibiotic therapy was tailored based on culture results from sputum, blood, urine, and wound samples. The diagnosis of inhalation injuries was facilitated through a combination of patient history, physical examinations, and fiberoptic bronchoscopy evaluation when necessary^12^.

### Data collection

Data were collected from the clinical data warehouse (CDW) of Hallym University Hangang Sacred Heart Hospital. The CDW is a centralized repository that integrates patient data from various sources, including electronic medical records, laboratory information systems, and pharmacy systems. The data collected included demographic information, burn injury characteristics (e.g. total body surface area burned (TBSA), burn depth, presence of inhalation injury), medical history, laboratory test results (including CBC tests), and outcomes (including AKI diagnosis based on AKIN criteria and mortality). CBC were collected upon admission, and subsequently, at 6 AM on the second day of admission. Depending on the patient's condition, multiple measurements could be taken within a single day. In cases where multiple measurements were conducted, the least favorable test results were selected for analysis.Blood samples were collected using the ethylenediamine tetraacetic acid (EDTA) tube, with 3 mL of blood drawn. Immediately following collection, the tube was gently inverted approximately 10 times for thorough mixing. All laboratory analyses were conducted within a two-hour window following sample collection to ensure the integrity of the results. In instances where the CBC results were deemed potentially erroneous based on clinical judgment, a retest was performed to confirm the findings. The diagnosis and classification of the severity of AKI were based on the AKIN criteria^4^. Stage 1 is defined by an increase in serum creatinine by ≥ 0.3 mg/dL (≥ 26.5 µmol/L) within 48 h or an increase to 1.5–1.9 times baseline within the prior 7 days, or urine output less than 0.5 mL/kg/h for 6–12 h. Stage 2 is characterized by an increase in serum creatinine to 2.0–2.9 times baseline within the prior 7 days, or urine output less than 0.5 mL/kg/h for more than 12 h. Stage 3 involves an increase in serum creatinine to 3.0 times baseline within the prior 7 days, or an increase in serum creatinine to ≥ 4.0 mg/dL (≥ 353.6 µmol/L), or initiation of renal replacement therapy, or, in patients younger than 18 years, a decrease in estimated glomerular filtration rate (eGFR) to less than 35 mL/min/1.73 m^2^, or urine output less than 0.3 mL/kg/h for more than 24 h or anuria for more than 12 h. Patients were classified into the highest stage they reached at any point during their hospital stay. The baseline serum creatinine was defined as the lowest value during hospital admission. If this was not available, the baseline was estimated using the Modification of Diet in Renal Disease (MDRD) equation assuming a baseline glomerular filtration rate of 75 mL/min/1.73 m^2^. The primary outcome was the in-hospital mortality rate within 60 days.

In addition to the above, it is important to note that Complete Blood Count (CBC) testing was routinely conducted as part of the ICU monitoring protocol, with samples collected daily. This ensured that we had comprehensive and timely data for each patient at all points leading up to the diagnosis of AKI. This continuous data collection was crucial in allowing us to detect and monitor changes relevant to AKI diagnosis in burn patients, thereby enhancing the accuracy and reliability of our findings.

### Definition of ratio markers

1. Neutrophil-to-lymphocyte ratio (NLR): The NLR is a measure of the balance between the number of neutrophils and lymphocytes in the blood. It is calculated by dividing the absolute neutrophil count by the absolute lymphocyte count. Elevated NLR values have been associated with poor outcomes in various conditions^13^.
2. Platelet-to-lymphocyte ratio (PLR): The PLR represents the ratio of the number of platelets to the number of lymphocytes. It is calculated by dividing the absolute platelet count by the absolute lymphocyte count. Similar to the NLR, a high PLR is associated with increased systemic inflammation and is indicative of a poorer prognosis for these diseases^14^.
3. Monocyte-to-lymphocyte ratio (MLR): The MLR is calculated by dividing the absolute monocyte count by the absolute lymphocyte count. An increased MLR has been observed to be associated with a poorer prognosis in diverse diseases^14^.
4. Systemic immune-inflammation index (SII): The SII is an index calculated using the counts of platelets, neutrophils, and lymphocytes. It is determined by multiplying the platelet count by the neutrophil count and dividing it by the lymphocyte count. Elevated SII values have been linked to postoperative patient conditions and poor prognoses in various diseases^15^.
5. MPV-to-platelet ratio (MPVPR), MPV-to-lymphocyte ratio (MPVLR): MPV-to-monocyte ratio (MPVMR), MPV-to-Neutrophil Ratio (MPVNR). The MPV ratio is calculated as the average platelet volume divided by the number of platelets, lymphocyte, monocyte and neutrophil.

Although there is limited evidence, some studies have shown the potential effectiveness of these markers^16^.

### Data management

Data were managed in accordance with the principles of good clinical practice and data protection regulations. Patient identifiers were removed to ensure patient confidentiality. The data were checked for completeness and consistency, and any discrepancies were resolved by referring back to the original source documents. Outliers were capped through a process known as Winsorization to reduce the impact of extreme values on the analysis. The data were then scaled to ensure that all variables were on a similar scale, thereby preventing any single variable from dominating the analysis.

### Statistical analysis

Statistical analyses were conducted using the R programming language (version 4.3.1). We have detailed the demographic characteristics in the following manner: continuous variables conforming to a normal distribution are described as mean values accompanied by their respective standard deviations (SD), while variables not following a normal distribution are reported using medians alongside their interquartile ranges (IQR). To compare the means of two groups, either an independent *t* test or a Wilcoxon signed-rank test was utilized, contingent on the distributional normality of the data. For comparisons involving more than two groups, Analysis of Variance (ANOVA) was employed, predicated on assumptions of normal distribution, independence, and equal variances across groups. In instances where these assumptions were not met, the non-parametric Kruskal–Wallis Test was applied as an alternative. For the evaluation of categorical variables, proportions were calculated and comparisons were executed using either the chi-square test or Fisher’s exact test, as dictated by the data distribution and sample size. In order to evaluate the diagnostic performance of AKI occurrence, regardless of the AKIN stage, several metrics including the Area Under the Curve (AUC), accuracy, sensitivity, specificity, positive predictive value (PPV), and negative predictive value (NPV) were calculated. The optimal cutoff point was determined using the Youden index. Performance score was calculated using the compare_performance function from the performance package. The score is a heuristic measure that ranges from 0 to 100%, with higher values indicating superior model performance.

For evaluating the AKIN classification, we utilized vector generalized linear and additive models (VGAM). This statistical method is particularly suited for modeling ordinal outcomes such as AKIN stages. We employed ordinal logistic regression models to assess the severity discrimination power across different AKIN stages. The primary focus of this analysis was not to predict mortality but to understand the impact of various severity markers on different AKIN stages. Mortality rates were examined through Cox proportional hazards models for each AKIN stage, which were also fitted using the VGAM package. Hazard ratios (HRs) were calculated to understand the impact of these markers on mortality. Additionally, the models were adjusted for age, TBSA, and inhalation injuries, as these are known significant predictors for burn patients. It should be noted that all statistical analyses were conducted exclusively on complete cases, eliminating the concern for missing data.

### Ethics approval and consent to participate

The study was conducted in accordance with the principles outlined in the Declaration of Helsinki. Approval was obtained from the Institutional Review Board of Hangang Sacred Heart Hospital (HG 2022-011). Informed consent from participants was waived due to the retrospective nature and design of the study.

## Results

### Demographics and characteristics of enrolled patients

**Figure 1:**
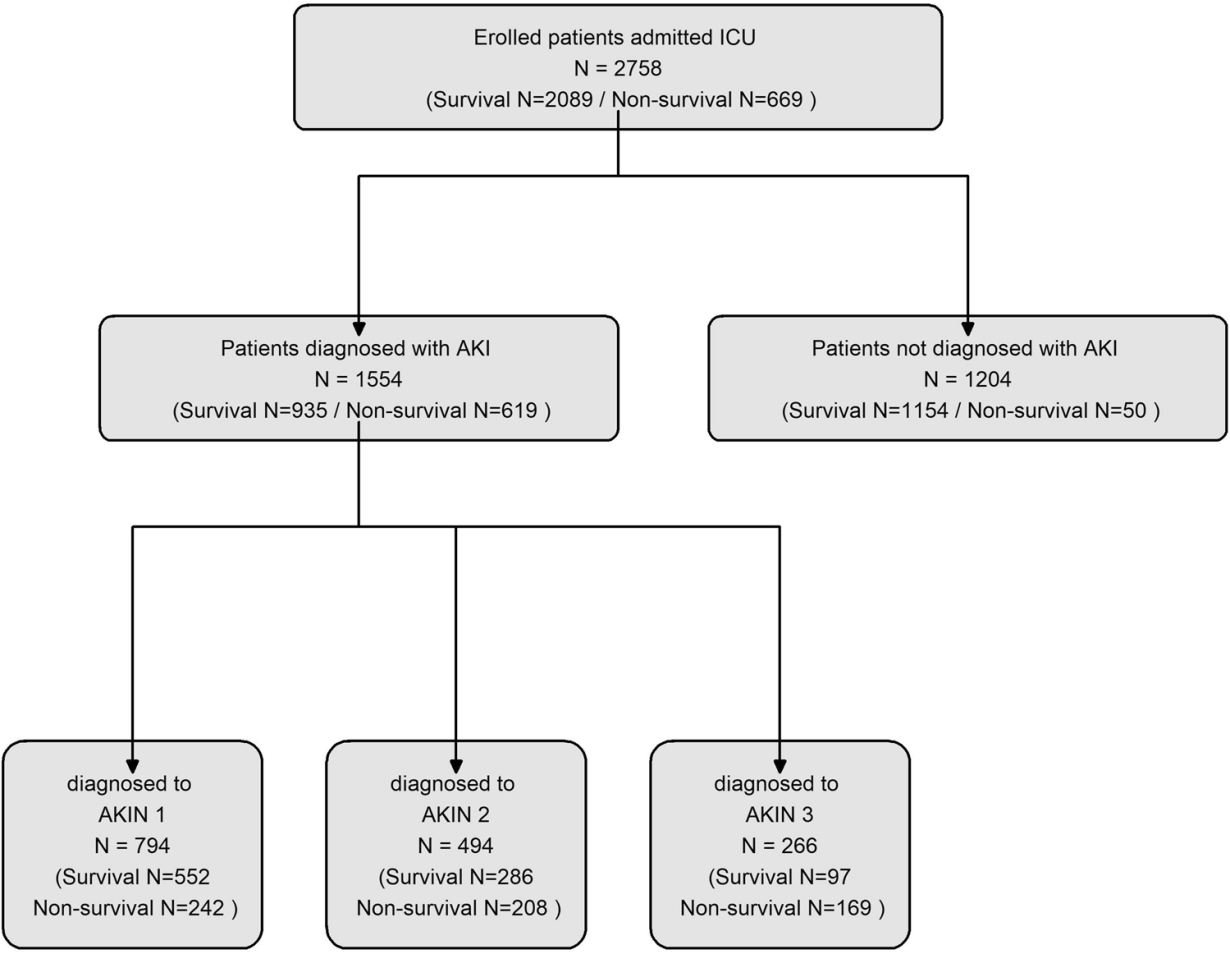
Flowchart depicting the enrollment process of patients in the study.

Among the 2758 patients included in the study, none had preexisting renal disease. Of these, 669 patients experienced mortality, resulting in a mortality rate of 24.3%. Additionally, acute kidney injury (AKI) occurred in 1554 patients, accounting for 56.3% of the study population. Based on the AKIN stage classification, 794 patients (28.8%) were categorized as AKIN 1, 494 patients (17.9%) as AKIN 2, and 266 patients (9.6%) as AKIN 3. The flowchart depicting the enrollment of patients is presented in Fig. 1. Figure S1 illustrates the frequency and proportion of AKI and AKIN grades by hospital day. Statistically significant differences were observed in all demographic and severity score variables when comparing patients with and without AKIN and AKI, except for gender. The median age of the 2181 patients was 52, with males accounting for the majority (79.1%). The median TBSA affected by burns was 27%, and inhalation burns occurred in 38.5% of the 1063 patients. All severity scores showed higher values as the AKIN stage increased, indicating a poorer prognosis for AKI. Significant differences were observed in CBC parameters and ratio markers between patients with different AKIN stages or AKI status, except for MPVMR. Detailed figures can be found in Table 1.

**Table 1:** Characteristics of enrolled patients by AKIN stage and AKI status.

Group	Variables	AKIN stage						AKI	
		Overall, N = 2758	AKIN_1, N = 794 (28.8%)	AKIN_2, N = 494 (17.9%)	AKIN_3, N = 266 (9.64%)	p-value	Yes, N = 1554 (56.3%)	No, N = 1204 (43.7%)	p-value
Demographics	Mortality	669 (24.3%)	242 (30.5%)	208 (42.1%)	169 (63.5%)	< 0.001	619 (39.8%)	50 (4.2%)	< 0.001
	Age					< 0.001			< 0.001
	Median [IQR]	52 [41, 63]	52 [41, 63]	54 [45, 66]	57 [46, 69]		53 [43, 66]	50 [39, 59]	
	Sex					0.312			0.813
	Male	2181 (79.1%)	629 (79.2%)	398 (80.6%)	199 (74.8%)		1226 (78.9%)	955 (79.3%)	
	Female	577 (20.9%)	165 (20.8%)	96 (19.4%)	67 (25.2%)		328 (21.1%)	249 (20.7%)	
	Type					< 0.001			< 0.001
	FB	1918 (69.6%)	605 (76.2%)	372 (75.3%)	193 (72.6%)		1170 (75.3%)	748 (62.2%)	
	SB	275 (10.0%)	77 (9.7%)	57 (11.5%)	33 (12.4%)		167 (10.7%)	108 (9.0%)	
	EB	369 (13.4%)	74 (9.3%)	30 (6.1%)	14 (5.3%)		118 (7.6%)	251 (20.9%)	
	ChB	46 (1.7%)	10 (1.3%)	5 (1.0%)	6 (2.3%)		21 (1.4%)	25 (2.1%)	
	CoB	148 (5.4%)	28 (3.5%)	30 (6.1%)	20 (7.5%)		78 (5.0%)	70 (5.8%)	
	TBSA					< 0.001			< 0.001
	Median [IQR]	27 [15, 46]	34 [20, 60]	33 [20, 60]	40 [20, 70]		35 [20, 60]	20 [10, 32]	
	Inhalation	1063 (38.5%)	365 (46.0%)	226 (45.7%)	120 (45.1%)	< 0.001	711 (45.8%)	352 (29.2%)	< 0.001
	LOS					< 0.001			< 0.001
	Median [IQR]	12 [5, 27]	22 [9, 37]	19 [7, 36]	11 [5, 23]		19 [7, 36]	7 [4, 17]	
Severity Scores*	Mortality	669 (24.3%)	242 (30.5%)	208 (42.1%)	169 (63.5%)	< 0.001	619 (39.8%)	50 (4.2%)	< 0.001
	ABSI					< 0.001			< 0.001
	Median [IQR]	8 [6, 10]	9 [7, 11]	9 [7, 11]	10 [7, 12]		9 [7, 11]	7 [5, 8]	
	rBaux					< 0.001			< 0.001
	Median [IQR]	88 [70, 110]	97 [77, 119]	101 [81, 122]	109 [85, 135]		100 [80, 123]	77 [62, 91]	
	Hangang					< 0.001			< 0.001
	Median [IQR]	125 [113, 142]	132 [120, 148]	137 [124, 156]	150 [134, 169]		136 [123, 155]	115 [108, 123]	
	APACHE IV					< 0.001			< 0.001
	Median [IQR]	36 [23, 60]	42 [29, 62]	49 [35, 71]	70 [48, 103]		48 [32, 71]	26 [19, 37]	
	SOFA					< 0.001			< 0.001
	Median [IQR]	3 [1, 4]	3 [2, 5]	4 [2, 6]	6 [4, 9]		4 [2, 6]	2 [0, 3]	
WBC related	WBC					< 0.001			< 0.001
	Median [IQR]	12.3 [8.5, 19.8]	16.7 [11.4, 23.9]	17.8 [11.7, 23.9]	16.6 [11.1, 23.9]		17.1 [11.5, 23.9]	9.3 [7.2, 12.0]	
	Neutrophil					< 0.001			< 0.001
	Median [IQR]	9.5 [6.2, 16.0]	13.8 [8.9, 19.7]	14.2 [9.0, 20.4]	13.5 [8.2, 20.4]		14.0 [8.8, 20.2]	6.8 [5.0, 9.4]	
	Lymphocyte					< 0.001			< 0.001
	Median [IQR]	1.36 [0.98, 2.00]	1.40 [0.94, 2.20]	1.54 [1.04, 2.57]	1.47 [0.87, 2.78]		1.46 [0.98, 2.41]	1.29 [0.97, 1.69]	
	Monocyte					< 0.001			< 0.001
	Median [IQR]	0.73 [0.50, 1.10]	0.82 [0.54, 1.30]	0.83 [0.55, 1.30]	0.79 [0.47, 1.31]		0.82 [0.53, 1.30]	0.66 [0.48, 0.89]	
	Eosinophil					< 0.001			< 0.001
	Median [IQR]	0.13 [0.08, 0.24]	0.11 [0.07, 0.20]	0.10 [0.06, 0.20]	0.10 [0.04, 0.20]		0.10 [0.06, 0.20]	0.18 [0.10, 0.29]	
	Basophil					< 0.001			< 0.001
	Median [IQR]	0.05 [0.03, 0.10]	0.07 [0.04, 0.10]	0.07 [0.03, 0.10]	0.06 [0.04, 0.10]		0.07 [0.04, 0.10]	0.04 [0.02, 0.06]	
	Immature granulocyte					< 0.001			< 0.001
	Median [IQR]	0.10 [0.04, 0.27]	0.16 [0.07, 0.40]	0.17 [0.07, 0.35]	0.24 [0.08, 1.05]		0.17 [0.08, 0.45]	0.06 [0.03, 0.12]	
RBC related	RBC					< 0.001			< 0.001
	Median [IQR]	4.20 [3.43, 5.12]	4.87 [3.72, 5.20]	4.89 [3.78, 5.20]	4.34 [3.52, 5.20]		4.81 [3.68, 5.20]	3.76 [3.28, 4.32]	
	RDW					< 0.001			< 0.001
	Median [IQR]	13.19 [12.39, 14.24]	13.19 [12.30, 14.24]	13.33 [12.60, 14.49]	14.00 [13.10, 15.62]		13.30 [12.54, 14.50]	13.00 [12.24, 13.75]	
	Hct					< 0.001			< 0.001
	Median [IQR]	42 [33, 46]	46 [35, 46]	46 [36, 46]	42 [33, 46]		45 [35, 46]	34 [30, 39]	
	Hb					< 0.001			< 0.001
	Median [IQR]	13.80 [10.50, 16.00]	15.30 [11.00, 16.60]	15.45 [11.38, 16.60]	13.80 [10.60, 16.00]		15.10 [11.00, 16.50]	11.20 [9.50, 12.93]	
	MCV					< 0.001			< 0.001
	Median [IQR]	91.7 [88.9, 94.7]	91.8 [88.8, 94.7]	92.8 [89.7, 95.7]	93.4 [90.6, 97.2]		92.3 [89.3, 95.5]	91.0 [88.5, 93.7]	
	MCH					< 0.001			< 0.001
	Median [IQR]	30.70 [29.70, 31.80]	30.80 [29.80, 31.90]	31.10 [30.10, 32.30]	31.00 [30.00, 32.70]		30.90 [29.90, 32.10]	30.50 [29.60, 31.50]	
	MCHC					< 0.001			< 0.001
	Median [IQR]	33.70 [32.90, 34.40]	33.80 [33.10, 34.60]	33.80 [32.80, 34.50]	33.50 [32.63, 34.30]		33.80 [32.90, 34.50]	33.60 [32.80, 34.30]	
Platelet related	Platelet					0.032			0.010
	Median [IQR]	258 [187, 357]	260 [190, 337]	258 [190, 345]	243 [151, 351]		258 [183, 342]	260 [190, 381]	
	MPV					< 0.001			< 0.001
	Median [IQR]	9.80 [9.24, 10.55]	9.84 [9.24, 10.64]	9.94 [9.30, 10.80]	10.40 [9.36, 11.33]		9.94 [9.30, 10.80]	9.70 [9.10, 10.30]	
	PDW					< 0.001			< 0.001
	Median [IQR]	11.31 [9.90, 13.59]	11.40 [10.00, 13.43]	11.85 [10.35, 14.43]	12.41 [10.59, 15.52]		11.68 [10.20, 14.00]	10.17 [9.00, 11.60]	
	PCT					0.004			< 0.001
	Median [IQR]	0.20 [0.18, 0.30]	0.20 [0.19, 0.30]	0.20 [0.17, 0.30]	0.20 [0.13, 0.33]		0.20 [0.18, 0.30]	0.21 [0.19, 0.30]	
Ratios	NLR					< 0.001			< 0.001
	Median [IQR]	8 [5, 13]	11 [7, 18]	12 [7, 17]	11 [7, 19]		11 [7, 18]	5 [4, 8]	
	PLR					0.011			0.023
	Median [IQR]	201 [134, 305]	203 [134, 311]	193 [132, 279]	186 [122, 285]		193 [130, 297]	208 [139, 314]	
	MLR					< 0.001			< 0.001
	Median [IQR]	0.58 [0.40, 0.89]	0.69 [0.44, 1.04]	0.68 [0.45, 1.00]	0.58 [0.38, 0.97]		0.67 [0.43, 1.00]	0.52 [0.37, 0.72]	
	SII					< 0.001			< 0.001
	Median [IQR]	1932 [1059, 3496]	2629 [1433, 4340]	2498 [1433, 4218]	2311 [1203, 4135]		2511 [1404, 4273]	1413 [876, 2332]	
	MPVPR					< 0.001			< 0.001
	Median [IQR]	0.04 [0.03, 0.06]	0.04 [0.03, 0.06]	0.05 [0.03, 0.07]	0.05 [0.03, 0.09]		0.05 [0.03, 0.07]	0.04 [0.03, 0.05]	
	MPVLR					< 0.001			< 0.001
	Median [IQR]	8.2 [5.8, 12.0]	9.0 [5.9, 13.7]	8.8 [5.7, 13.4]	9.3 [5.5, 15.6]		9.0 [5.8, 13.7]	7.6 [5.8, 10.4]	
	MPVMR					0.030			0.965
	Median [IQR]	15 [10, 22]	15 [10, 23]	15 [10, 23]	17 [10, 30]		15 [10, 23]	15 [11, 21]	
	MPVNR					< 0.001			< 0.001
	Median [IQR]	1.16 [0.74, 1.73]	0.90 [0.60, 1.41]	0.89 [0.60, 1.45]	0.94 [0.60, 1.63]		0.90 [0.60, 1.47]	1.44 [1.05, 1.99]	

*FB* flame burn, *SB* scale burn, *EB* electrical burn, *ChB* chemical burn, *CoB* contact burn, *LOS* length of hospital stay, *ABSI* abbreviated burn severity index, *SOFA* sequential organ failure assessment.

*All severity scores were calculated at the time of diagnosis.

### Diagnostic performance of CBC parameters and ratios for predicting AKI

**Figure 2:**
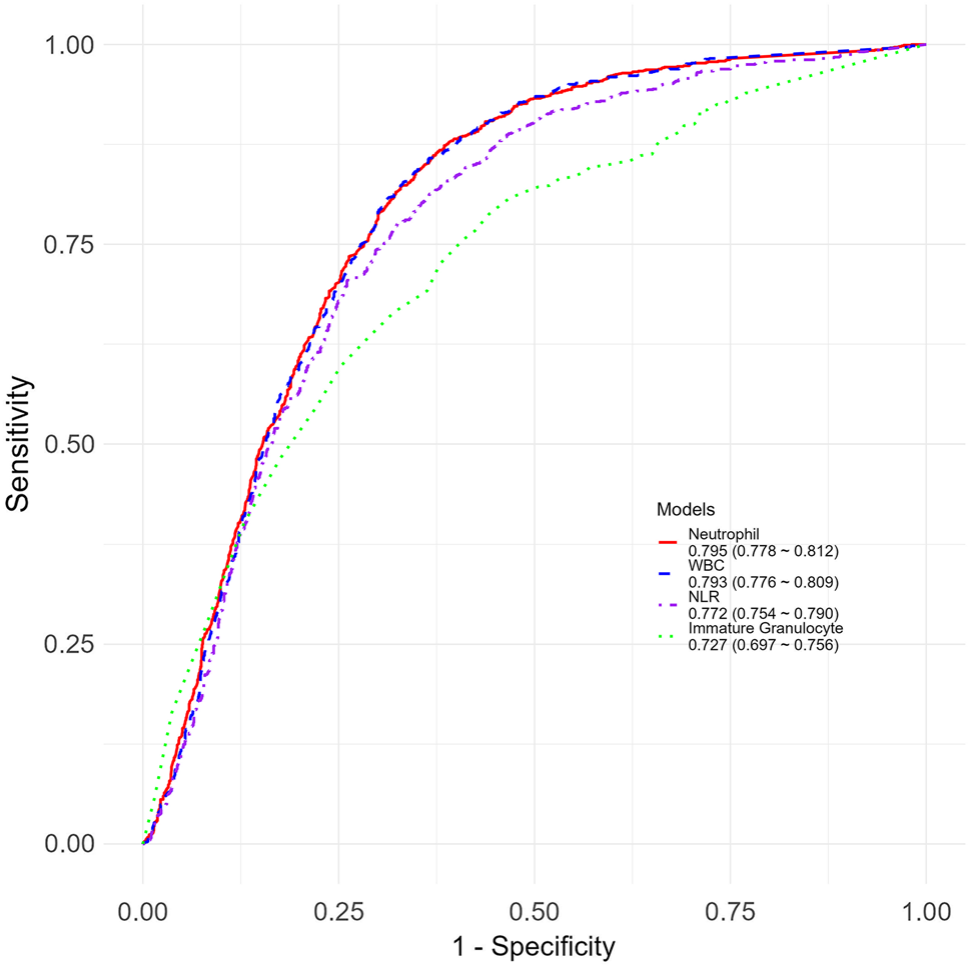
Top four area under the curve (AUC) values for predicting acute kidney injury (AKI).

Among the white blood cell (WBC)-related parameters, the AUC with 95% Confidence Interval (CI) values for neutrophil count and WBC count were 0.790 (0.778–0.812) and 0.793 (0.776–0.809), respectively, followed by immature granulocyte count with an AUC of 0.727 (0.697–0.756). For red blood cell (RBC)-related parameters, the AUC values for hematocrit (Hct), hemoglobin (Hb), and RBC count were 0.725 (0.706–0.744), 0.713 (0.694–0.732), and 0.713 (0.693–0.732), respectively. Among the platelet-related parameters, only platelet distribution width (PDW) had an AUC of 0.677 (0.657–0.698). Among the ratio markers, the NLR had the highest AUC at 0.772 (0.754–0.790), followed by MPVNR and SII with AUC values of 0.700 (0.680–0.719) and 0.680 (0.660–0.700), respectively. Figure 2 presents the top 4 variables with the highest AUC values, including neutrophil count, WBC count, NLR, and immature granulocyte count. The AUC values and performance metrics for all variables are presented in Table S1.

### Severity discrimination power of AKIN stage

**Figure 3:**
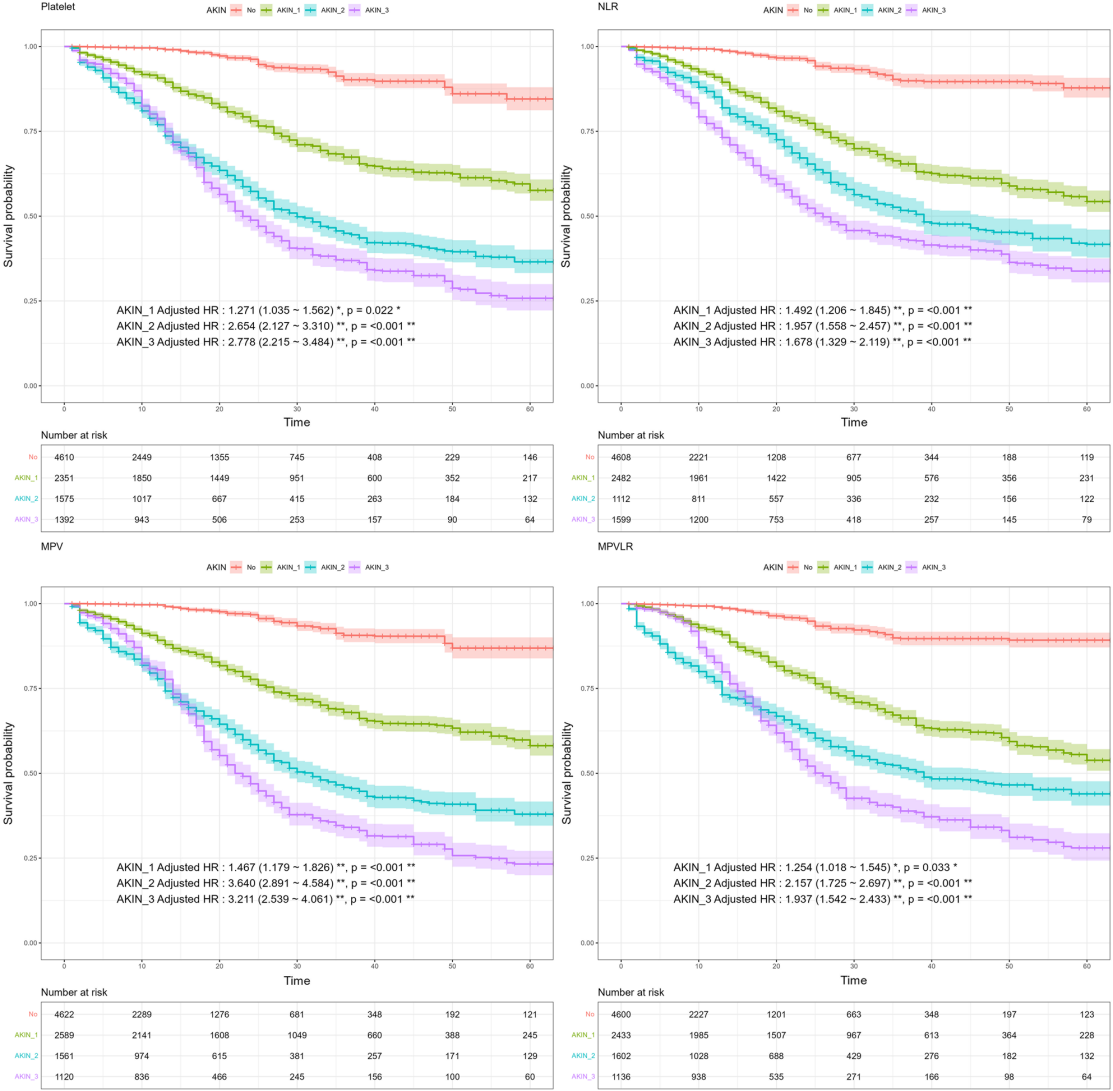
Adjusted hazard ratios (HR) for AKIN stages in predicting outcomes.

Adjusted odds ratios (ORs) from the ordinal logistic regression are detailed in Table S2, indicating the relative likelihood of higher AKIN stages. Furthermore, certain markers displayed significant hazard ratios (HRs) for mortality across all AKIN stages 1, 2, and 3. Specifically, the markers Platelet, MPV, NLR, and MPVLR all showed HR values greater than or equal to 1. For Platelet, the HR values were 1.271, 2.654, and 2.778 in AKIN stages 1, 2, and 3, respectively. For MPV, the corresponding HR values were 1.467, 3.640, and 3.211; for NLR, they were 1.492, 1.957, and 1.678; and for MPVLR, the HR values were 1.254, 2.157, and 1.937. These findings are illustrated in Fig. 3. Complete HR values by AKIN stage for all markers are presented in Table S3. Figure S2 outlines the time-dependent variations of all CBC parameters according to AKIN stage.

## Discussions

Our investigation aimed to determine the predictive validity of routine CBC tests in predicting AKI and assessing AKIN severity in burn patients. The AKI is a prevalent complication in severe burn patients, resulting in increased mortality, morbidity, and hospitalization duration^17^. Reliable early prediction and assessment tools are therefore essential for prompt intervention and optimal patient management. We focused on CBC markers, particularly those related to WBC and platelet-lymphocyte dynamics, hypothesizing that they could be critical in predicting AKI and determining its severity. CBC tests provide information about three types of cells: RBC, WBC, and platelets. In burn patients, significant changes in WBC count, particularly neutrophils and lymphocytes, as well as platelet counts are frequently observed due to a systemic inflammatory response (SIRS) and other inflammatory complications^18–20^.

Inflammation-induced AKI, such as that observed in severe burn cases, occurs due to a complex interplay between inflammatory cells, cytokines, and the renal vasculature, leading to endothelial damage and renal dysfunction^21^. Our study revealed a significant correlation between WBC count, neutrophil count and PLR with AKI incidence and severity in burn patients. High WBC count and PLR were associated with increased AKI incidence and platelet and MPV were associated with AKIN stages, aligning with previous research suggesting that leukocytosis and platelet activation could contribute to kidney injury^22–24^.

The relationship between WBC count, PLR, and AKI may be partly attributed to the role of these cells in the inflammatory response. Neutrophils and platelets release pro-inflammatory mediators, promoting endothelial damage, oxidative stress, and capillary leakage^25^. Similarly, a decreased lymphocyte count, indicated by high PLR, reflects an imbalance in the immune response that could exacerbate tissue injury^26^. These findings have hold potential for future clinical applications. Routinely performed CBC tests could serve as an initial screening tool for renal dysfunction in burn patients. These preliminary results could then inform the decision to proceed with more specialized and potentially expensive tests for further evaluation, enabling early intervention and potentially reducing associated morbidity and mortality.

Future research endeavors should prioritize the validation of our findings by extending the scope of investigation across diverse cohorts and multiple healthcare facilities. The immediate objective of our forthcoming studies will be to focus intently on this indispensable step of validation to establish both the broader applicability and credibility of our results.

However, our study has certain limitations. The generalizability of our findings is constrained due to the single-center nature of our study and its focus on ICU admissions, which may not fully represent the broader population of burn patients. The retrospective design also leaves room for selection bias and confounding factors. While we attempted to mitigate these limitations by analyzing temporal trends in WBC and PLR, it’s important to note that these associations do not imply causality. Additionally, the reliability of CBC tests can be impacted by individual variations, as well as errors in sample collection, processing, and interpretation.

Further research should also consider evaluating other inflammatory markers, including serum phosphate levels, in combination with CBC tests to enhance predictive accuracy. Serum phosphate levels have been reported as significant in AKI, reflecting renal function impairment and muscle breakdown, particularly in the context of burn injuries. Integrating serum phosphate levels with CBC markers could provide a more comprehensive assessment of AKI risk in burn patients, combining insights into renal function and systemic inflammatory response.

Despite these limitations, we believe our study has the potential to contribute to the field of AKI research by proposing the use of routine CBC tests, particularly WBC count and PLR, as effective tools for predicting AKI, and platelet, MPV, and NLR as indicators for mortality prediction in burn patients with AKI. Our findings highlight the untapped potential of easily accessible clinical data for improving patient management, while also emphasizing the necessity of integrating both clinical and laboratory data for more precise medical care. We encourage further studies to validate these preliminary findings and to solidify their clinical relevance.

Additionally, we acknowledge the limitations in immediately applying our approach of using the lowest creatinine level during hospitalization as the baseline for diagnosing AKI in clinical practice. It is more pragmatic to use the most recent creatinine level that reflects the patient’s current status and to continuously monitor changes in renal function. In cases where the lowest creatinine level is not available, we use the MDRD equation to estimate the baseline creatinine level. This method is particularly useful when there is insufficient information about the patient’s previous renal function, offering a practical approach to estimating the baseline creatinine level in a clinical setting. The decision to use the AKIN criteria for our study was influenced by its proven sensitivity in burn patients, as indicated in Chung’s research^27^. The AKIN criteria’s flexibility in not requiring a fixed baseline serum creatinine is particularly beneficial in burn care, where determining a baseline can be challenging due to the dynamic nature of the patient’s condition and treatment. This flexibility, combined with the criteria’s focus on changes in serum creatinine within specific time frames, aligns well with the needs of acute burn care and enhances our ability to accurately assess AKI in this context.

In conclusion, our findings support the potential utility of routine CBC tests for diagnosing and assessing the severity of AKI in burn patients. WBC count and PLR show promise as predictive markers for AKI, while platelet, MPV, and NLR can help assess its severity. Further validation through multicenter studies is needed for broader applicability.

### Supplementary Information

Supplementary Information.

## Data Availability

The datasets used and/or analyzed during the current study are available from the corresponding author upon reasonable request.
