# Supplementary material for: Pioneering predictions of AKI and AKIN severity in burn patients: a comprehensive CBC approach
Source: Sci Rep. 2024 Jan 5;14:675. doi: 10.1038/s41598-024-51253-x (PMC10770361; doi:10.1038/s41598-024-51253-x)

Supplementary material

Pioneering Predictions of AKI and AKIN Severity in Burn Patients: A Comprehensive CBC Approach

Jongsoo Park1, Dohern Kym,1,2*, Myongjin Kim1, Yong Suk Cho 1,2, Jun Hur1,2, Wook Chun1,2, Dogeon Yoon2, Jaechul Yoon1

1 Department of Surgery and Critical Care, Burn Center, Hangang Sacred Heart Hospital, Hallym University Medical Center, 12, Beodeunaru-ro 7-gil, Youngdeungpo-gu, Seoul, Korea, 07247

2 Burn Institutes, Hangang Sacred Heart Hospital, Hallym University Medical Center, 12, Beodeunaru-ro 7-gil, Youngdeungpo-gu, Seoul, Korea, 07247

*Corresponding authors

Department of Surgery and Critical Care, Burn Center, Hangang Sacred Heart Hospital, College of Medicine, Hallym University 12, Beodeunaru-ro 7-gil, Youngdeungpo-gu, Seoul, Korea, 07247

Tel. 82-2-2639-5446, Fax. 82-2-2678-4386, E-mail: [dohern@hallym.or.kr](mailto:dohern@hallym.or.kr)

Contents

[STROBE Statement—Checklist of items that should be included in reports of cohort studies 4](#_Toc151918235)

[Table S1. Performance of CBC parameters Predicting AKI: AUC Analysis 7](#_Toc151918236)

[Table S2. Odds Ratios for AKIN Severity Predictions using VGAM 11](#_Toc151918237)

[Table S3. Adjusted HR for Mortality using VGAM Model in Enrolled Patients 15](#_Toc151918238)

[Figure S1. Frequency and Proportion of Acute Kidney Injury (AKI) and AKI Network (AKIN) Grades by Hospital Day 17](#_Toc151918239)

[Figure S2. Repaeted Measures over Time 18](#_Toc151918240)

[1) WBC 18](#_Toc151918241)

[2) Neutrophil 19](#_Toc151918242)

[3) Lymphocyte 20](#_Toc151918243)

[4) Monocyte 21](#_Toc151918244)

[5) Eosinophil 22](#_Toc151918245)

[6) Basophil 23](#_Toc151918246)

[7) Immature Granulocyte 24](#_Toc151918247)

[8) RBC 25](#_Toc151918248)

[9) RDW 26](#_Toc151918249)

[10) Hct 27](#_Toc151918250)

[11) Hb 28](#_Toc151918251)

[12 MCV 29](#_Toc151918252)

[13) MCH 30](#_Toc151918253)

[14) MCHC 31](#_Toc151918254)

[15) Platelet 32](#_Toc151918255)

[16) MPV 33](#_Toc151918256)

[17) PDW 34](#_Toc151918257)

[18) PCT 35](#_Toc151918258)

[19) NLR 36](#_Toc151918259)

[20 PLR 37](#_Toc151918260)

[21 MLR 38](#_Toc151918261)

[22) SII 39](#_Toc151918262)

[23) MPVPR 40](#_Toc151918263)

[24) MPVLR 41](#_Toc151918264)

[25) MPVMR 42](#_Toc151918265)

[26) MPVNR 43](#_Toc151918266)

# STROBE Statement—Checklist of items that should be included in reports of cohort studies

|  | | Item No | | Recommendation | Checked | |  |
| --- | --- | --- | --- | --- | --- | --- | --- |
| **Title and abstract** | | 1 | | (*a*) Indicate the study’s design with a commonly used term in the title or the abstract | Yes | |  |
|  |  |  |  | (*b*) Provide in the abstract an informative and balanced summary of what was done and what was found | Yes | |  |
| Introduction | | | | | | |  |
| Background/rationale | | 2 | | Explain the scientific background and rationale for the investigation being reported | Yes | |  |
| Objectives | | 3 | | State specific objectives, including any prespecified hypotheses | Yes | |  |
| Methods | | | | | | |  |
| Study design | | 4 | | Present key elements of study design early in the paper | Yes | |  |
| Setting | | 5 | | Describe the setting, locations, and relevant dates, including periods of recruitment, exposure, follow-up, and data collection | Yes | |  |
| Participants | | 6 | | (*a*) Give the eligibility criteria, and the sources and methods of selection of participants. Describe methods of follow-up | Yes | |  |
|  |  |  |  | (*b*) For matched studies, give matching criteria and number of exposed and unexposed | Yes | |  |
| Variables | | 7 | | Clearly define all outcomes, exposures, predictors, potential confounders, and effect modifiers. Give diagnostic criteria, if applicable | Yes | |  |
| Data sources/ measurement | | 8* | | For each variable of interest, give sources of data and details of methods of assessment (measurement). Describe comparability of assessment methods if there is more than one group | Yes | |  |
| Bias | | 9 | | Describe any efforts to address potential sources of bias | Yes | |  |
| Study size | | 10 | | Explain how the study size was arrived at | No | |  |
| Quantitative variables | | 11 | | Explain how quantitative variables were handled in the analyses. If applicable, describe which groupings were chosen and why | Yes | |  |
| Statistical methods | | 12 | | (*a*) Describe all statistical methods, including those used to control for confounding | Yes | |  |
|  |  |  |  | (*b*) Describe any methods used to examine subgroups and interactions | Yes | |  |
|  |  |  |  | (*c*) Explain how missing data were addressed | Yes | |  |
|  |  |  |  | (*d*) If applicable, explain how loss to follow-up was addressed | NA | |  |
|  |  |  |  | (*e*) Describe any sensitivity analyses | Yes | |  |
| Results | | | | |  | |  |
| Participants | | 13* | | (a) Report numbers of individuals at each stage of study—eg numbers potentially eligible, examined for eligibility, confirmed eligible, included in the study, completing follow-up, and analysed | Yes | |  |
|  |  |  |  | (b) Give reasons for non-participation at each stage | NA | |  |
|  |  |  |  | (c) Consider use of a flow diagram | Yes | |  |
| Descriptive data | | 14* | | (a) Give characteristics of study participants (eg demographic, clinical, social) and information on exposures and potential confounders | Yes | |  |
|  |  |  |  | (b) Indicate number of participants with missing data for each variable of interest | Yes | |  |
|  |  |  |  | (c) Summarise follow-up time (eg, average and total amount) | NA | |  |
| Outcome data | | 15* | | Report numbers of outcome events or summary measures over time | Yes | |  |
| Main results | 16 | | (*a*) Give unadjusted estimates and, if applicable, confounder-adjusted estimates and their precision (eg, 95% confidence interval). Make clear which confounders were adjusted for and why they were included | | | Yes | |
|  |  |  | (*b*) Report category boundaries when continuous variables were categorized | | | Yes | |
|  |  |  | (*c*) If relevant, consider translating estimates of relative risk into absolute risk for a meaningful time period | | | Yes | |
| Other analyses | 17 | | Report other analyses done—eg analyses of subgroups and interactions, and sensitivity analyses | | | Yes | |
| Discussion | | | | | | | |
| Key results | 18 | | Summarise key results with reference to study objectives | | | Yes | |
| Limitations | 19 | | Discuss limitations of the study, taking into account sources of potential bias or imprecision. Discuss both direction and magnitude of any potential bias | | | Yes | |
| Interpretation | 20 | | Give a cautious overall interpretation of results considering objectives, limitations, multiplicity of analyses, results from similar studies, and other relevant evidence | | | Yes | |
| Generalisability | 21 | | Discuss the generalisability (external validity) of the study results | | | Yes | |
| Other information | | | | | | | |
| Funding | 22 | | Give the source of funding and the role of the funders for the present study and, if applicable, for the original study on which the present article is based | | | Yes | |

*Give information separately for exposed and unexposed groups.

**Note:** An Explanation and Elaboration article discusses each checklist item and gives methodological background and published examples of transparent reporting. The STROBE checklist is best used in conjunction with this article (freely available on the Web sites of PLoS Medicine at http://www.plosmedicine.org/, Annals of Internal Medicine at http://www.annals.org/, and Epidemiology at http://www.epidem.com/). Information on the STROBE Initiative is available at http://www.strobe-statement.org.

# Table S1. Performance of CBC parameters Predicting AKI: AUC Analysis

| CBC parameters | Variables | AUC (95% CI) | Optimal cut-off | Accurarcy (95% CI) | Sensitivity (95% CI) | Specificity (95% CI) | PPV (95% CI) | NPV (95% CI) |
| --- | --- | --- | --- | --- | --- | --- | --- | --- |
| WBC-realated | WBC | 0.793 (0.776 ~ 0.809) | 12.62 | 0.731 (0.714 ~ 0.748) | 0.727 (0.704 ~ 0.749) | 0.737 (0.711 ~ 0.762) | 0.779 (0.757 ~ 0.801) | 0.679 (0.653 ~ 0.704) |
|  | Neutrophil | 0.795 (0.778 ~ 0.812) | 10.29 | 0.732 (0.714 ~ 0.748) | 0.714 (0.690 ~ 0.736) | 0.754 (0.729 ~ 0.778) | 0.784 (0.761 ~ 0.805) | 0.678 (0.652 ~ 0.703) |
|  | Lymphocyte | 0.578 (0.557 ~ 0.599) | 2.25 | 0.536 (0.517 ~ 0.555) | 0.698 (0.674 ~ 0.721) | 0.333 (0.307 ~ 0.361) | 0.567 (0.544 ~ 0.590) | 0.468 (0.435 ~ 0.503) |
|  | Monocyte | 0.616 (0.595 ~ 0.637) | 0.96 | 0.568 (0.549 ~ 0.587) | 0.703 (0.679 ~ 0.726) | 0.399 (0.371 ~ 0.427) | 0.594 (0.571 ~ 0.617) | 0.518 (0.485 ~ 0.550) |
|  | Eosinophil | 0.619 (0.598 ~ 0.641) | 0.14 | 0.582 (0.562 ~ 0.601) | 0.814 (0.792 ~ 0.834) | 0.308 (0.282 ~ 0.335) | 0.581 (0.559 ~ 0.603) | 0.584 (0.544 ~ 0.623) |
|  | Basophil | 0.682 (0.661 ~ 0.703) | 0.05 | 0.640 (0.621 ~ 0.659) | 0.670 (0.644 ~ 0.695) | 0.603 (0.574 ~ 0.632) | 0.676 (0.651 ~ 0.701) | 0.596 (0.567 ~ 0.625) |
|  | Immature Granulocyte | 0.727 (0.697 ~ 0.756) | 0.12 | 0.627 (0.598 ~ 0.655) | 0.394 (0.353 ~ 0.437) | 0.852 (0.820 ~ 0.880) | 0.721 (0.666 ~ 0.771) | 0.592 (0.558 ~ 0.626) |
| RBC-realated | RBC | 0.713 (0.693 ~ 0.732) | 4.66 | 0.640 (0.621 ~ 0.658) | 0.709 (0.686 ~ 0.732) | 0.551 (0.523 ~ 0.579) | 0.669 (0.645 ~ 0.692) | 0.597 (0.568 ~ 0.626) |
|  | RDW | 0.594 (0.573 ~ 0.615) | 13.60 | 0.572 (0.553 ~ 0.591) | 0.773 (0.751 ~ 0.794) | 0.315 (0.289 ~ 0.342) | 0.590 (0.569 ~ 0.612) | 0.521 (0.484 ~ 0.557) |
|  | Hct | 0.725 (0.706 ~ 0.744) | 42.90 | 0.646 (0.628 ~ 0.664) | 0.729 (0.706 ~ 0.751) | 0.539 (0.510 ~ 0.568) | 0.673 (0.650 ~ 0.695) | 0.605 (0.575 ~ 0.635) |
|  | Hb | 0.713 (0.694 ~ 0.732) | 14.10 | 0.633 (0.615 ~ 0.651) | 0.697 (0.674 ~ 0.720) | 0.550 (0.522 ~ 0.579) | 0.668 (0.644 ~ 0.691) | 0.584 (0.554 ~ 0.613) |
|  | MCV | 0.583 (0.562 ~ 0.604) | 92.40 | 0.560 (0.541 ~ 0.579) | 0.815 (0.794 ~ 0.834) | 0.234 (0.211 ~ 0.259) | 0.576 (0.555 ~ 0.597) | 0.497 (0.455 ~ 0.539) |
|  | MCH | 0.575 (0.554 ~ 0.596) | 31.40 | 0.551 (0.532 ~ 0.569) | 0.844 (0.825 ~ 0.862) | 0.176 (0.155 ~ 0.199) | 0.567 (0.546 ~ 0.587) | 0.469 (0.422 ~ 0.516) |
|  | MCHC | 0.545 (0.523 ~ 0.566) | 33.60 | 0.546 (0.527 ~ 0.565) | 0.952 (0.940 ~ 0.962) | 0.027 (0.019 ~ 0.038) | 0.556 (0.536 ~ 0.575) | 0.308 (0.223 ~ 0.405) |
| Platelet-realated | Platelet | 0.529 (0.507 ~ 0.551) | 358.00 | 0.562 (0.544 ~ 0.581) | 0.928 (0.914 ~ 0.940) | 0.096 (0.080 ~ 0.114) | 0.567 (0.548 ~ 0.587) | 0.509 (0.442 ~ 0.576) |
|  | MPV | 0.583 (0.561 ~ 0.604) | 10.14 | 0.558 (0.539 ~ 0.577) | 0.797 (0.776 ~ 0.817) | 0.253 (0.229 ~ 0.278) | 0.577 (0.555 ~ 0.598) | 0.494 (0.454 ~ 0.535) |
|  | PDW | 0.677 (0.657 ~ 0.698) | 11.00 | 0.650 (0.632 ~ 0.668) | 0.735 (0.712 ~ 0.757) | 0.540 (0.511 ~ 0.568) | 0.674 (0.651 ~ 0.696) | 0.611 (0.581 ~ 0.641) |
|  | PCT | 0.541 (0.520 ~ 0.563) | 0.21 | 0.561 (0.542 ~ 0.580) | 0.929 (0.915 ~ 0.941) | 0.093 (0.077 ~ 0.111) | 0.566 (0.546 ~ 0.585) | 0.507 (0.439 ~ 0.574) |
| Ratios | NLR | 0.772 (0.754 ~ 0.790) | 8.18 | 0.718 (0.701 ~ 0.735) | 0.696 (0.672 ~ 0.720) | 0.746 (0.720 ~ 0.770) | 0.774 (0.751 ~ 0.796) | 0.662 (0.637 ~ 0.688) |
|  | PLR | 0.526 (0.505 ~ 0.548) | 196.36 | 0.556 (0.537 ~ 0.575) | 1.000 (0.998 ~ 1.000) | 0.000 (0.000 ~ 0.003) | 0.556 (0.537 ~ 0.575) | NaN (0.000 ~ 1.000) |
|  | MLR | 0.618 (0.597 ~ 0.639) | 0.75 | 0.587 (0.568 ~ 0.606) | 0.700 (0.676 ~ 0.723) | 0.446 (0.417 ~ 0.474) | 0.612 (0.589 ~ 0.636) | 0.543 (0.511 ~ 0.574) |
|  | SII | 0.680 (0.660 ~ 0.700) | 1,881.85 | 0.654 (0.636 ~ 0.672) | 0.676 (0.652 ~ 0.700) | 0.627 (0.599 ~ 0.654) | 0.694 (0.670 ~ 0.717) | 0.608 (0.580 ~ 0.635) |
|  | MPVPR | 0.618 (0.597 ~ 0.639) | 0.03 | 0.603 (0.584 ~ 0.621) | 0.786 (0.765 ~ 0.806) | 0.369 (0.341 ~ 0.397) | 0.614 (0.592 ~ 0.635) | 0.575 (0.539 ~ 0.610) |
|  | MPVLR | 0.572 (0.551 ~ 0.594) | 12.70 | 0.533 (0.514 ~ 0.552) | 0.716 (0.693 ~ 0.739) | 0.304 (0.279 ~ 0.331) | 0.563 (0.540 ~ 0.585) | 0.462 (0.427 ~ 0.498) |
|  | MPVMR | 0.499 (0.477 ~ 0.520) | 26.59 | 0.555 (0.537 ~ 0.574) | 0.999 (0.996 ~ 1.000) | 0.001 (0.000 ~ 0.005) | 0.556 (0.537 ~ 0.574) | 0.500 (0.013 ~ 0.987) |
|  | MPVNR | 0.700 (0.680 ~ 0.719) | 0.91 | 0.598 (0.580 ~ 0.617) | 0.828 (0.807 ~ 0.846) | 0.312 (0.286 ~ 0.339) | 0.600 (0.579 ~ 0.622) | 0.591 (0.552 ~ 0.630) |
| The darkest color represents an AUC > 0.75. ; The middle color represents an AUC > 0.70. ; The lightest color represents an AUC > 0.65 | | | | | | | | |

# Table S2. Odds Ratios for AKIN Severity Predictions using VGAM

| CBC parameters | Variables | AKIN 1   adjusted OR (95%CI) | AKIN 1   adjusted OR (95%CI) over time | AKIN 2   adjusted OR (95%CI) | AKIN 2   adjusted OR (95%CI) over time | AKIN 3   adjusted OR (95%CI) | AKIN 3   adjusted OR (95%CI) over time | Performace Score |
| --- | --- | --- | --- | --- | --- | --- | --- | --- |
| WBC-realated | WBC | 1.343 (1.305 - 1.382) ** | 1.141 (1.110 - 1.174) ** | 1.199 (1.163 - 1.235) ** | 1.082 (1.049 - 1.115) ** | 1.069 (1.026 - 1.114) * | 1.051 (1.008 - 1.095) * | 0.64 |
|  | Neutrophil | 1.380 (1.341 - 1.421) ** | 1.147 (1.115 - 1.180) ** | 1.215 (1.178 - 1.252) ** | 1.084 (1.052 - 1.117) ** | 1.095 (1.050 - 1.141) ** | 1.043 (1.001 - 1.087) * | 0.62 |
|  | Lymphocyte | 0.969 (0.943 - 0.997) * | 1.089 (1.059 - 1.121) ** | 1.012 (0.981 - 1.044) | 1.077 (1.043 - 1.112) ** | 0.929 (0.889 - 0.971) * | 1.073 (1.025 - 1.124) * | 0.88 |
|  | Monocyte | 1.107 (1.077 - 1.138) ** | 1.061 (1.032 - 1.090) ** | 1.058 (1.026 - 1.090) ** | 1.050 (1.019 - 1.083) * | 0.982 (0.943 - 1.022) | 0.995 (0.955 - 1.038) | 0.85 |
|  | Eosinophil | 0.915 (0.890 - 0.941) ** | 0.954 (0.928 - 0.981) ** | 0.915 (0.884 - 0.946) ** | 0.999 (0.966 - 1.033) | 0.936 (0.891 - 0.982) * | 1.026 (0.977 - 1.079) | 0.87 |
|  | Basophil | 1.242 (1.204 - 1.280) ** | 1.038 (1.007 - 1.069) * | 1.106 (1.070 - 1.143) ** | 1.031 (0.998 - 1.065) | 1.025 (0.979 - 1.072) | 1.040 (0.995 - 1.087) | 0.80 |
|  | Immature Granulocyte | 1.430 (1.368 - 1.495) ** | 0.982 (0.941 - 1.024) | 1.232 (1.180 - 1.286) ** | 1.009 (0.969 - 1.051) | 1.185 (1.123 - 1.249) ** | 1.068 (1.016 - 1.123) * | 0.83 |
| RBC-realated | RBC | 1.117 (1.086 - 1.149) ** | 1.206 (1.172 - 1.240) ** | 1.034 (1.002 - 1.067) * | 1.102 (1.068 - 1.137) ** | 0.938 (0.898 - 0.980) * | 1.023 (0.978 - 1.069) | 0.77 |
|  | RDW | 1.186 (1.152 - 1.221) ** | 0.908 (0.883 - 0.934) ** | 1.203 (1.165 - 1.242) ** | 0.939 (0.910 - 0.968) ** | 1.303 (1.248 - 1.361) ** | 0.939 (0.900 - 0.980) * | 0.77 |
|  | Hct | 1.128 (1.095 - 1.162) ** | 1.215 (1.180 - 1.252) ** | 1.059 (1.025 - 1.094) ** | 1.114 (1.078 - 1.151) ** | 0.993 (0.950 - 1.039) | 1.056 (1.010 - 1.104) * | 0.69 |
|  | Hb | 1.103 (1.070 - 1.138) ** | 1.231 (1.194 - 1.269) ** | 1.032 (0.998 - 1.067) | 1.124 (1.087 - 1.163) ** | 0.962 (0.919 - 1.008) | 1.053 (1.005 - 1.103) * | 0.64 |
|  | MCV | 1.149 (1.118 - 1.181) ** | 1.008 (0.981 - 1.036) | 1.185 (1.148 - 1.222) ** | 1.024 (0.993 - 1.057) | 1.287 (1.232 - 1.345) ** | 1.005 (0.961 - 1.050) | 0.85 |
|  | MCH | 1.105 (1.075 - 1.136) ** | 1.063 (1.034 - 1.092) ** | 1.117 (1.083 - 1.152) ** | 1.056 (1.024 - 1.090) ** | 1.195 (1.144 - 1.248) ** | 0.988 (0.946 - 1.032) | 0.88 |
|  | MCHC | 1.022 (0.995 - 1.050) | 1.080 (1.052 - 1.110) ** | 0.960 (0.932 - 0.990) * | 1.041 (1.010 - 1.073) * | 0.966 (0.927 - 1.006) | 0.976 (0.937 - 1.017) | 0.88 |
| Platelet-realated | Platelet | 0.799 (0.777 - 0.821) ** | 0.960 (0.935 - 0.987) * | 0.834 (0.809 - 0.860) ** | 1.011 (0.979 - 1.043) | 0.780 (0.744 - 0.818) ** | 1.122 (1.068 - 1.179) ** | 0.50 |
|  | MPV | 1.319 (1.282 - 1.358) ** | 0.979 (0.951 - 1.007) | 1.274 (1.236 - 1.314) ** | 0.955 (0.926 - 0.984) * | 1.262 (1.211 - 1.315) ** | 0.957 (0.919 - 0.997) * | 0.86 |
|  | PDW | 1.360 (1.320 - 1.403) ** | 1.062 (1.031 - 1.094) ** | 1.301 (1.261 - 1.342) ** | 0.984 (0.955 - 1.015) | 1.244 (1.193 - 1.297) ** | 0.953 (0.915 - 0.993) * | 0.80 |
|  | PCT | 0.837 (0.814 - 0.860) ** | 0.941 (0.916 - 0.966) ** | 0.877 (0.850 - 0.904) ** | 0.994 (0.963 - 1.025) | 0.840 (0.802 - 0.880) ** | 1.105 (1.053 - 1.159) ** | 0.70 |
| Ratios | NLR | 1.479 (1.434 - 1.526) ** | 1.105 (1.072 - 1.139) ** | 1.248 (1.212 - 1.285) ** | 1.017 (0.988 - 1.047) | 1.209 (1.164 - 1.256) ** | 0.989 (0.953 - 1.027) | 0.50 |
|  | PLR | 0.962 (0.936 - 0.988) * | 0.976 (0.950 - 1.002) | 0.935 (0.908 - 0.964) ** | 0.986 (0.957 - 1.017) | 0.912 (0.874 - 0.952) ** | 1.043 (0.998 - 1.090) | 0.86 |
|  | MLR | 1.223 (1.190 - 1.257) ** | 1.047 (1.019 - 1.076) ** | 1.117 (1.085 - 1.150) ** | 1.020 (0.990 - 1.051) | 1.087 (1.045 - 1.131) ** | 0.975 (0.937 - 1.014) | 0.86 |
|  | SII | 1.167 (1.136 - 1.199) ** | 1.062 (1.034 - 1.091) ** | 1.056 (1.025 - 1.087) ** | 1.051 (1.020 - 1.084) * | 0.978 (0.938 - 1.020) | 1.085 (1.039 - 1.133) ** | 0.80 |
|  | MPVPR | 1.248 (1.216 - 1.280) ** | 1.005 (0.981 - 1.029) | 1.297 (1.286 - 1.309) ** | 0.988 (0.979 - 0.997) * | 1.459 (1.441 - 1.477) ** | 0.946 (0.934 - 0.958) ** | 0.50 |
|  | MPVLR | 1.330 (1.290 - 1.372) ** | 1.004 (0.974 - 1.035) | 1.200 (1.165 - 1.235) ** | 0.953 (0.925 - 0.981) * | 1.213 (1.170 - 1.259) ** | 0.954 (0.919 - 0.989) * | 0.86 |
|  | MPVMR | 1.181 (1.146 - 1.217) ** | 0.993 (0.965 - 1.021) | 1.120 (1.087 - 1.153) ** | 0.969 (0.942 - 0.997) * | 1.174 (1.131 - 1.218) ** | 0.993 (0.959 - 1.029) | 0.86 |
|  | MPVNR | 0.910 (0.886 - 0.935) ** | 0.945 (0.920 - 0.971) ** | 0.943 (0.915 - 0.972) ** | 0.943 (0.915 - 0.972) ** | 1.000 (0.961 - 1.041) | 0.954 (0.917 - 0.992) * | 0.86 |
| ** This is p-vaue < 0.001.; * This is p-vaue < 0.05 | | | | | | | | |
| The darkest color represents an AUC > 0.75.; The middle color represents an AUC > 0.70.; The lightest color represents an AUC > 0.65 | | | | | | | | |

# Table S3. Adjusted HR for Mortality using VGAM Model in Enrolled Patients

| CBC parameters | Variables | AKIN_1   Adjusted Hazard Ratio (95%CI) | AKIN_2   Adjusted Hazard Ratio (95%CI) | AKIN_3   Adjusted Hazard Ratio (95%CI) |
| --- | --- | --- | --- | --- |
| WBC-realated | WBC | 0.743 (0.608 ~ 0.908) * | 1.222 (0.989 ~ 1.509) | 0.912 (0.732 ~ 1.137) |
|  | Neutrophil | 0.848 (0.692 ~ 1.038) | 1.282 (1.034 ~ 1.590) * | 0.922 (0.739 ~ 1.151) |
|  | Lymphocyte | 0.716 (0.589 ~ 0.870) ** | 1.132 (0.921 ~ 1.392) | 1.018 (0.815 ~ 1.272) |
|  | Monocyte | 0.618 (0.507 ~ 0.753) ** | 1.140 (0.923 ~ 1.408) | 0.953 (0.768 ~ 1.182) |
|  | Eosinophil | 0.644 (0.524 ~ 0.793) ** | 1.208 (0.968 ~ 1.507) | 0.954 (0.761 ~ 1.197) |
|  | Basophil | 0.606 (0.491 ~ 0.747) ** | 1.026 (0.822 ~ 1.281) | 0.832 (0.658 ~ 1.053) |
|  | Immature Granulocyte | 0.972 (0.733 ~ 1.290) | 1.021 (0.763 ~ 1.366) | 1.089 (0.806 ~ 1.472) |
| RBC-realated | RBC | 0.540 (0.447 ~ 0.653) ** | 1.018 (0.826 ~ 1.254) | 0.615 (0.504 ~ 0.751) ** |
|  | RDW | 0.963 (0.773 ~ 1.200) | 1.821 (1.441 ~ 2.300) ** | 2.493 (1.993 ~ 3.119) ** |
|  | Hct | 0.597 (0.497 ~ 0.717) ** | 1.167 (0.961 ~ 1.417) | 0.727 (0.597 ~ 0.885) * |
|  | Hb | 0.605 (0.503 ~ 0.727) ** | 1.118 (0.916 ~ 1.365) | 0.774 (0.638 ~ 0.939) * |
|  | MCV | 0.709 (0.584 ~ 0.861) ** | 1.459 (1.179 ~ 1.805) ** | 1.333 (1.083 ~ 1.640) * |
|  | MCH | 0.683 (0.564 ~ 0.826) ** | 1.243 (1.009 ~ 1.532) * | 1.131 (0.910 ~ 1.406) |
|  | MCHC | 0.695 (0.571 ~ 0.845) ** | 1.012 (0.817 ~ 1.253) | 0.967 (0.781 ~ 1.198) |
| Platelet-realated | Platelet | 1.271 (1.035 ~ 1.562) * | 2.654 (2.127 ~ 3.310) ** | 2.778 (2.215 ~ 3.484) ** |
|  | MPV | 1.467 (1.179 ~ 1.826) ** | 3.640 (2.891 ~ 4.584) ** | 3.211 (2.539 ~ 4.061) ** |
|  | PDW | 1.206 (0.986 ~ 1.473) | 2.747 (2.229 ~ 3.387) ** | 2.209 (1.770 ~ 2.756) ** |
|  | PCT | 1.010 (0.822 ~ 1.241) | 1.941 (1.555 ~ 2.423) ** | 2.010 (1.602 ~ 2.522) ** |
| Ratios | NLR | 1.492 (1.206 ~ 1.845) ** | 1.957 (1.558 ~ 2.457) ** | 1.678 (1.329 ~ 2.119) ** |
|  | PLR | 0.690 (0.566 ~ 0.840) ** | 1.244 (1.006 ~ 1.539) * | 1.041 (0.829 ~ 1.307) |
|  | MLR | 0.889 (0.729 ~ 1.085) | 1.312 (1.057 ~ 1.629) * | 1.054 (0.847 ~ 1.312) |
|  | SII | 0.623 (0.512 ~ 0.759) ** | 0.902 (0.726 ~ 1.119) | 0.831 (0.670 ~ 1.031) |
|  | MPVPR | 1.032 (0.825 ~ 1.290) | 2.847 (2.259 ~ 3.586) ** | 3.382 (2.695 ~ 4.243) ** |
|  | MPVLR | 1.254 (1.018 ~ 1.545) * | 2.157 (1.725 ~ 2.697) ** | 1.937 (1.542 ~ 2.433) ** |
|  | MPVMR | 0.793 (0.652 ~ 0.965) * | 1.243 (1.006 ~ 1.536) * | 1.263 (1.018 ~ 1.567) * |
|  | MPVNR | 0.665 (0.546 ~ 0.811) ** | 0.998 (0.809 ~ 1.232) | 0.902 (0.721 ~ 1.129) |
| ** This is p-vaue < 0.001.; * This is p-vaue < 0.05 | | | | |
| The darker color represents an HR > 1 with a p-value < 0.05 in all three sections.; The lighter color indicates an HR > 1 with a p-value < 0.05 in two sections | | | | |

# Figure S1. Frequency and Proportion of Acute Kidney Injury (AKI) and AKI Network (AKIN) Grades by Hospital Day


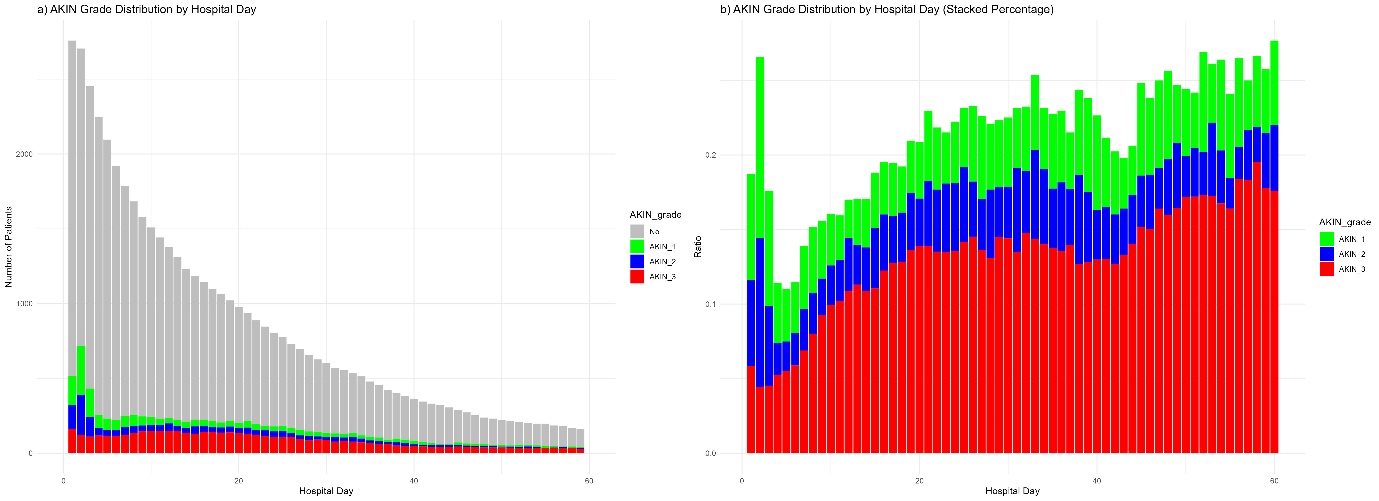


# Figure S2. Repaeted Measures over Time

## 1) WBC


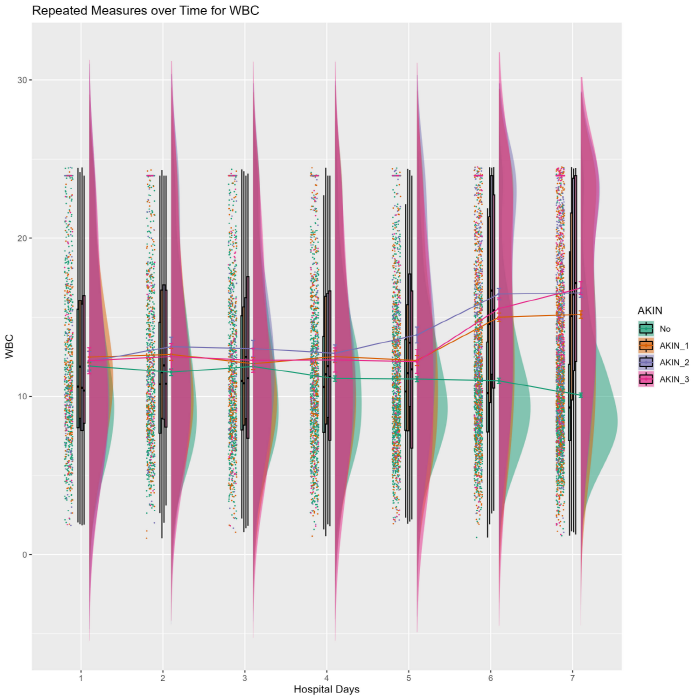


## 2) Neutrophil


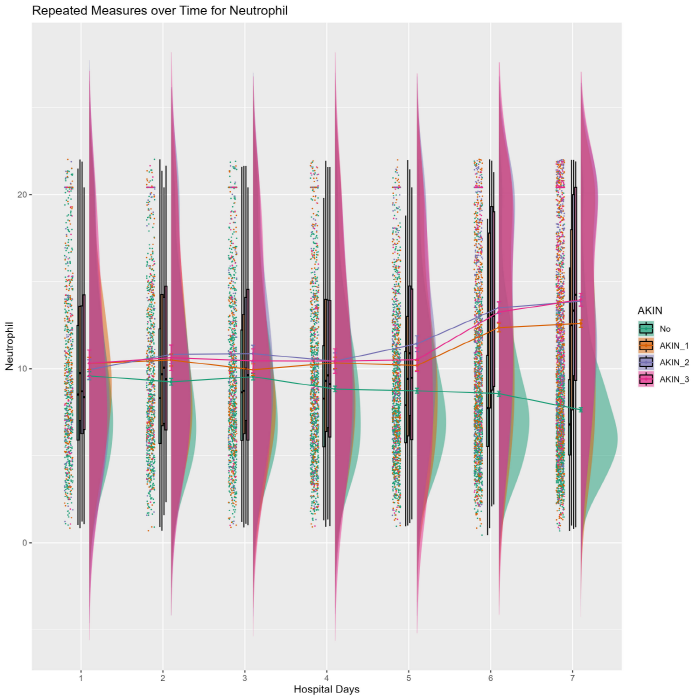


## 3) Lymphocyte


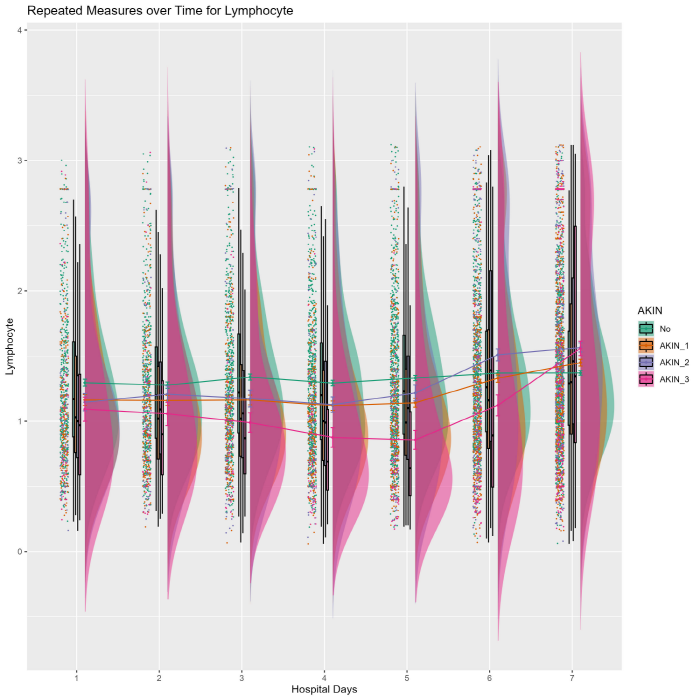


## 4) Monocyte


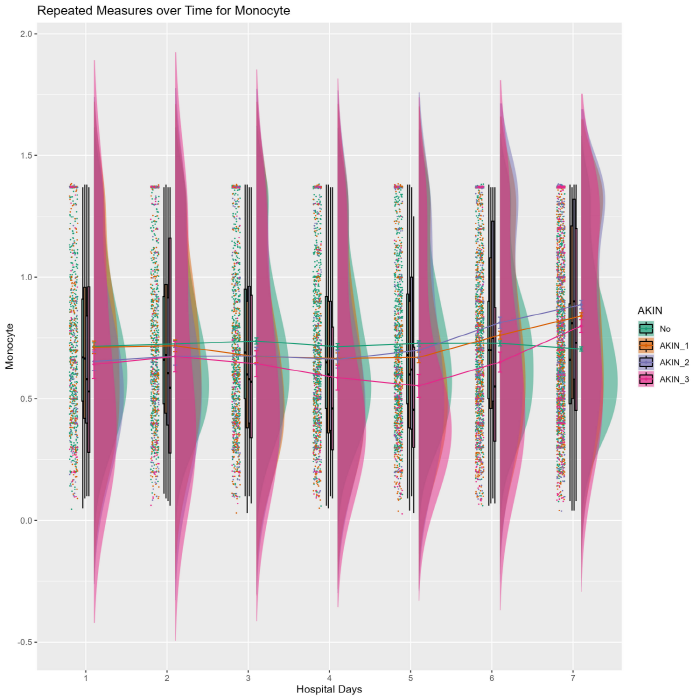


## 5) Eosinophil


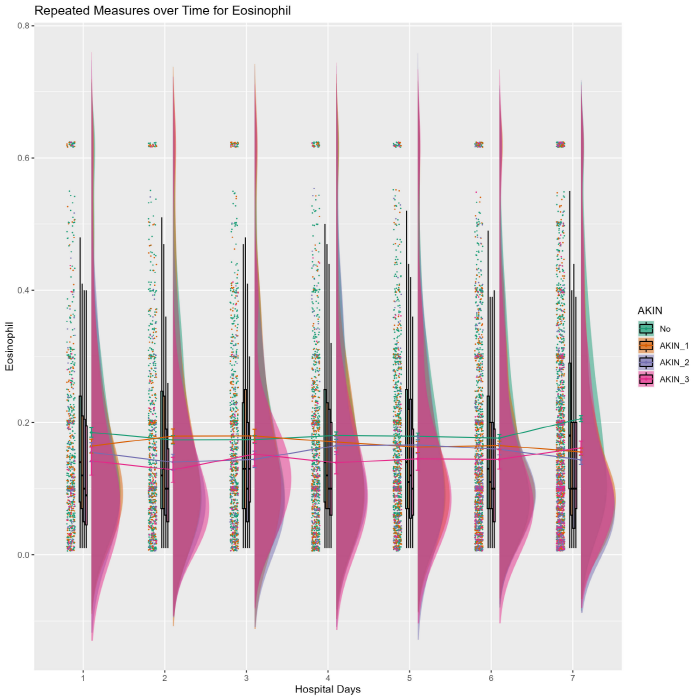


## 6) Basophil


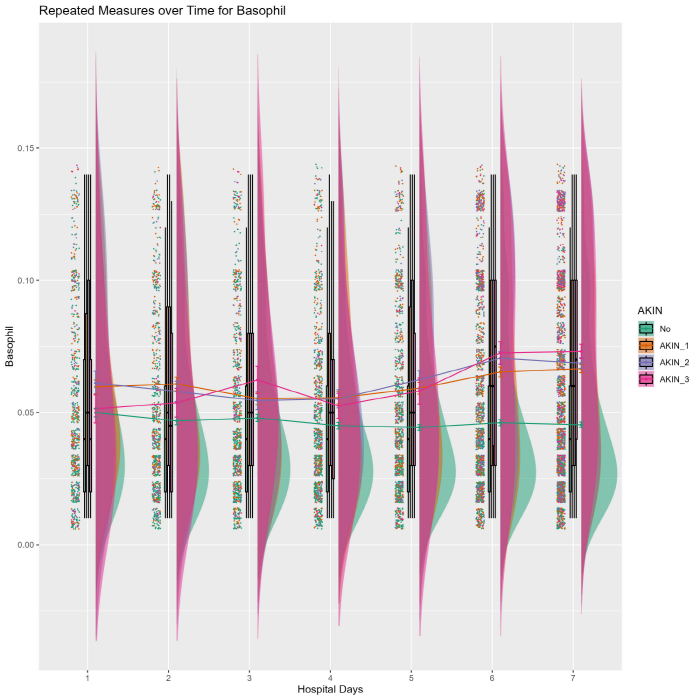


## 7) Immature Granulocyte


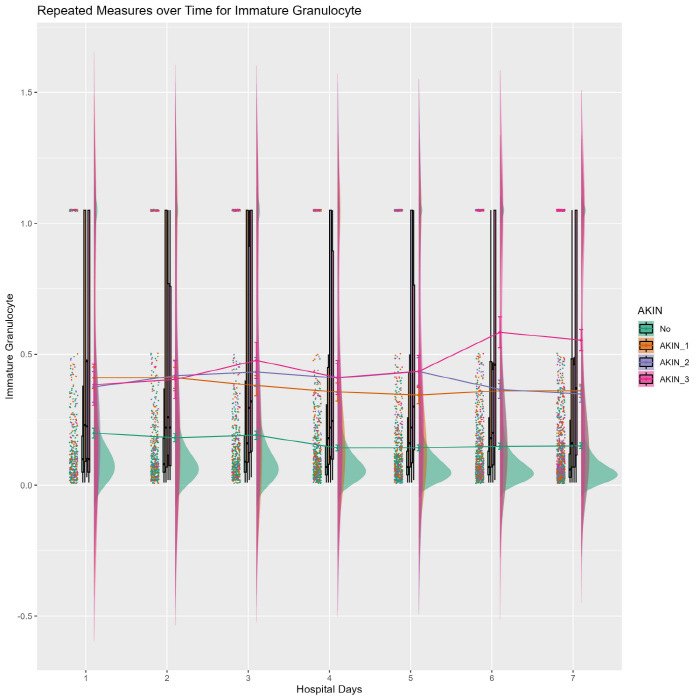


## 8) RBC


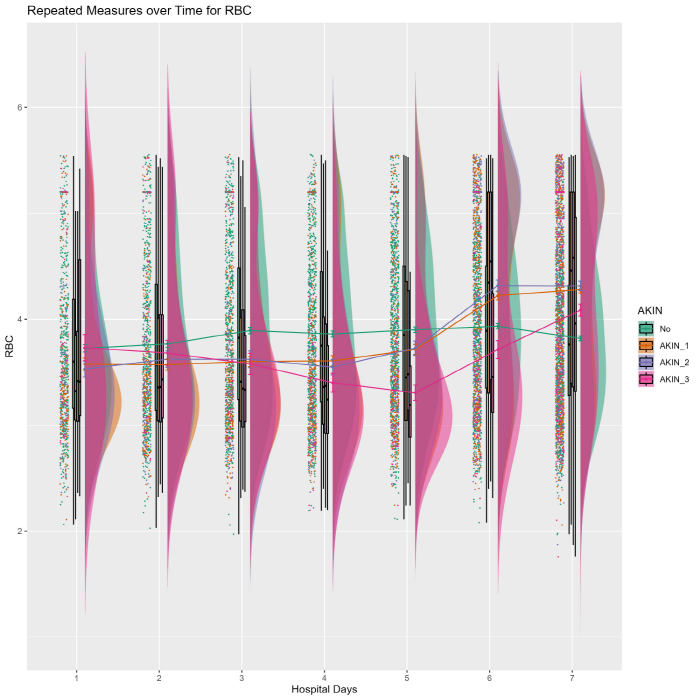


## 9) RDW


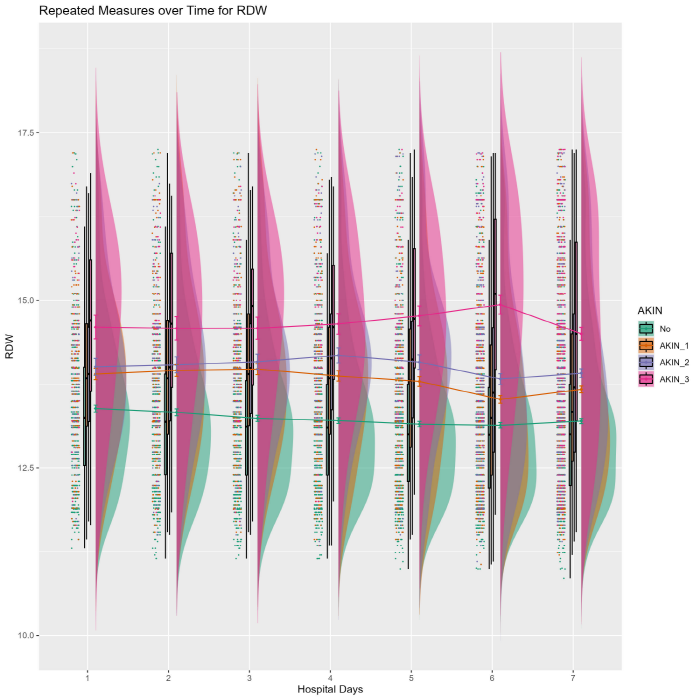


## 10) Hct


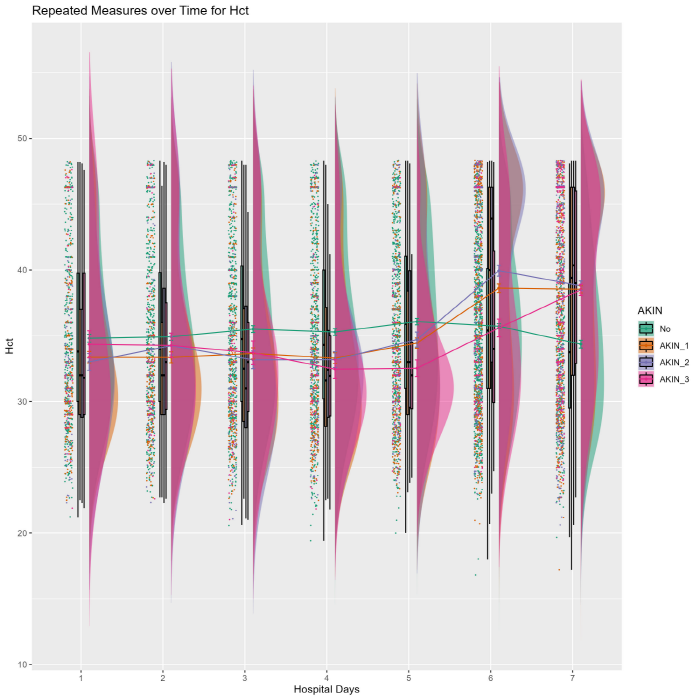


## 11) Hb


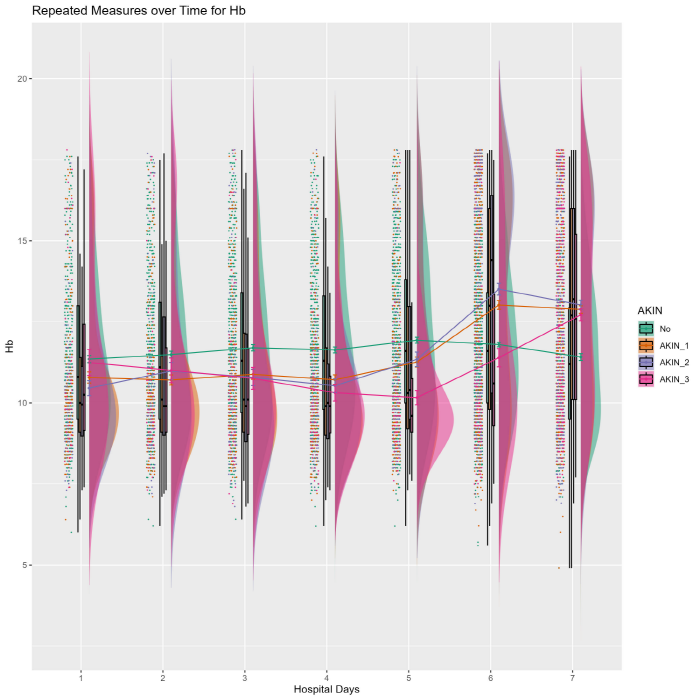


## 12 MCV


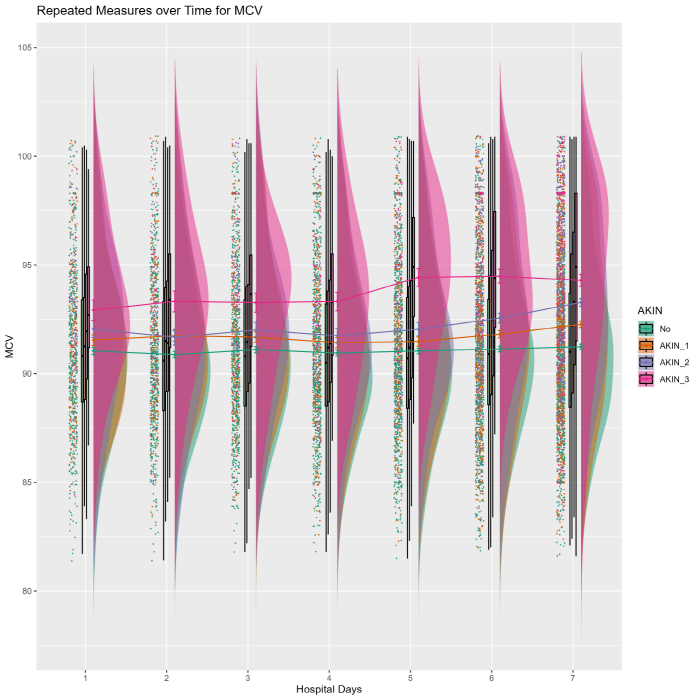


## 13) MCH


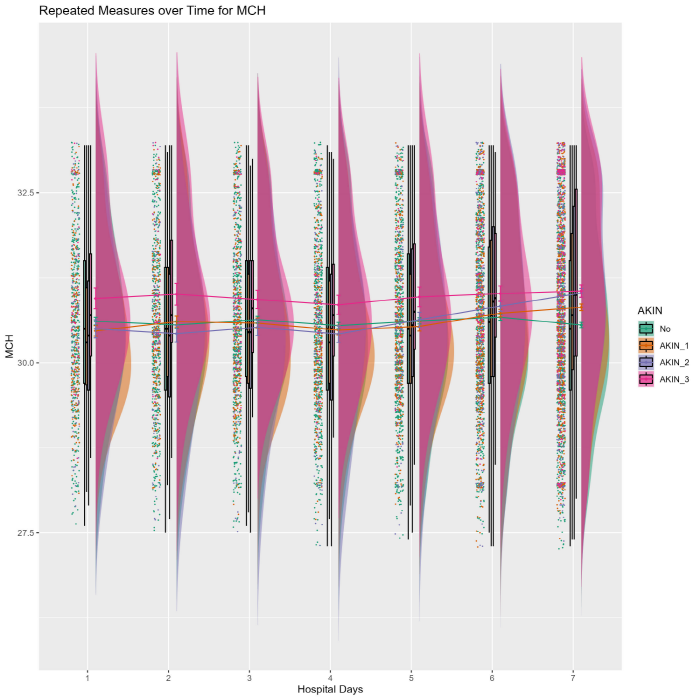


## 14) MCHC


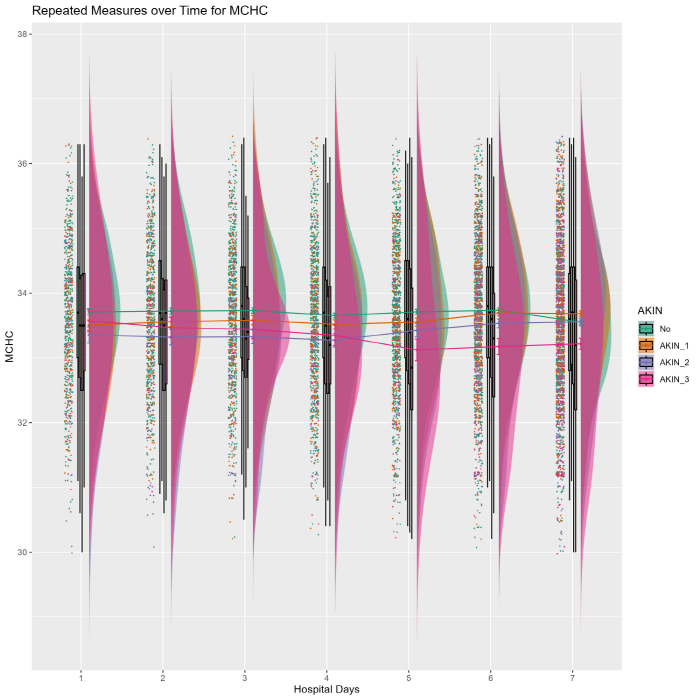


## 15) Platelet


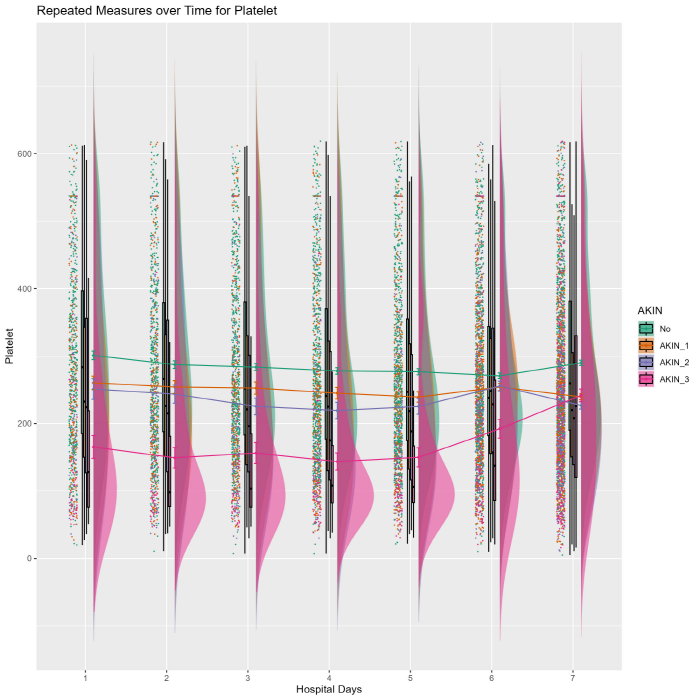


## 16) MPV


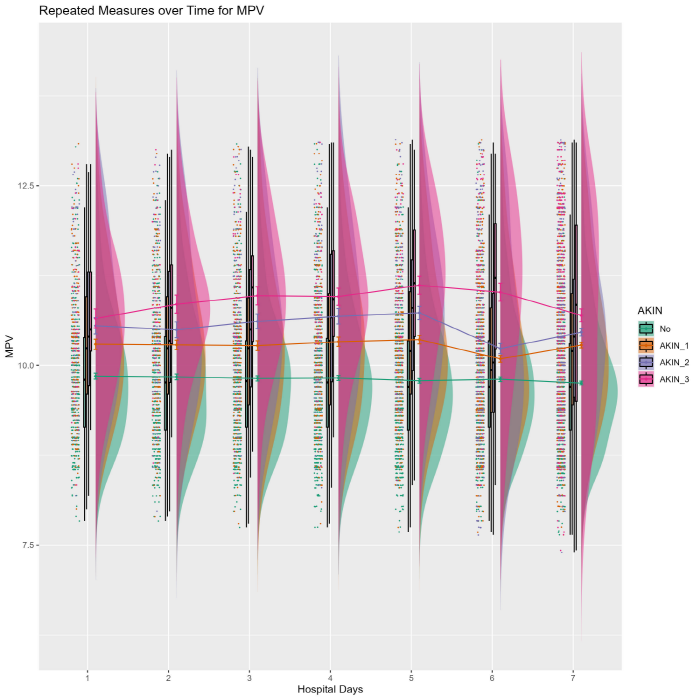


## 17) PDW


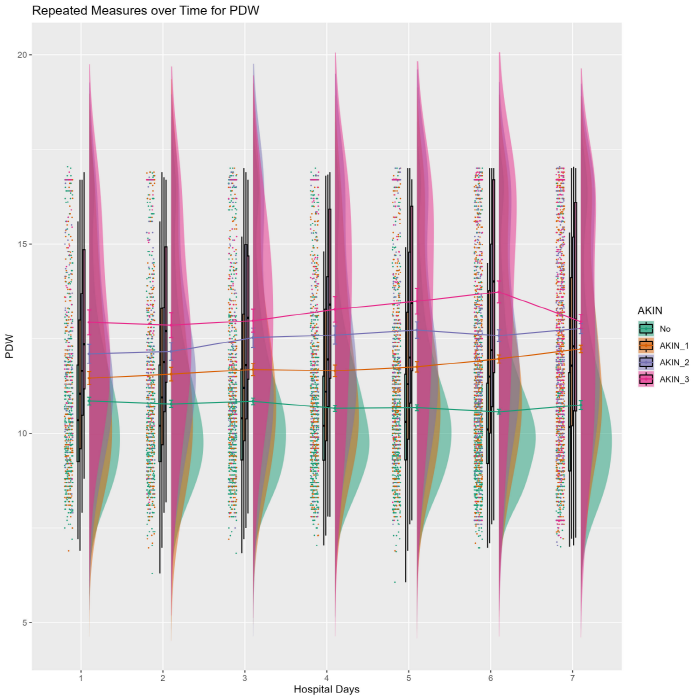


## 18) PCT


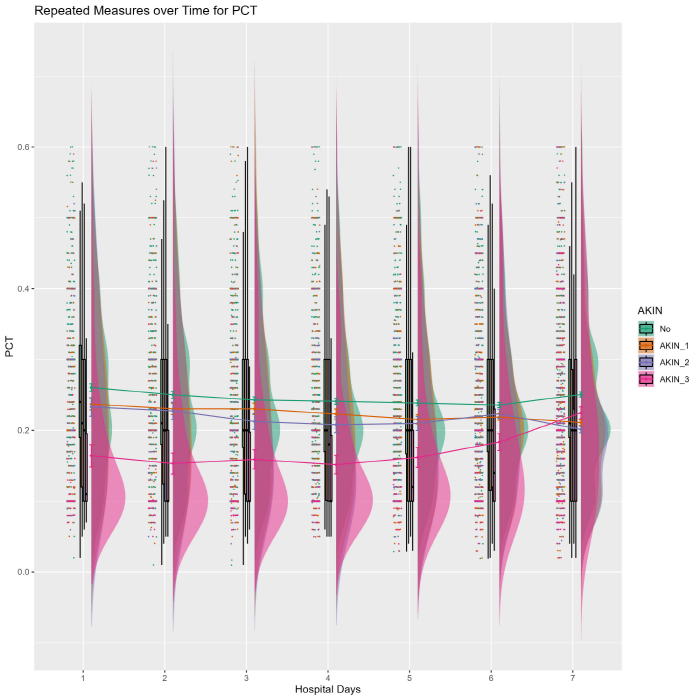


## 19) NLR


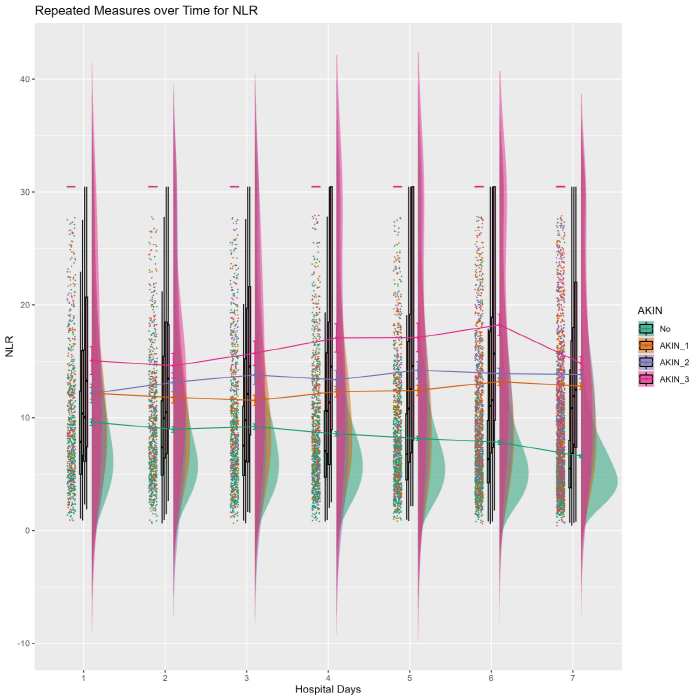


## 20 PLR


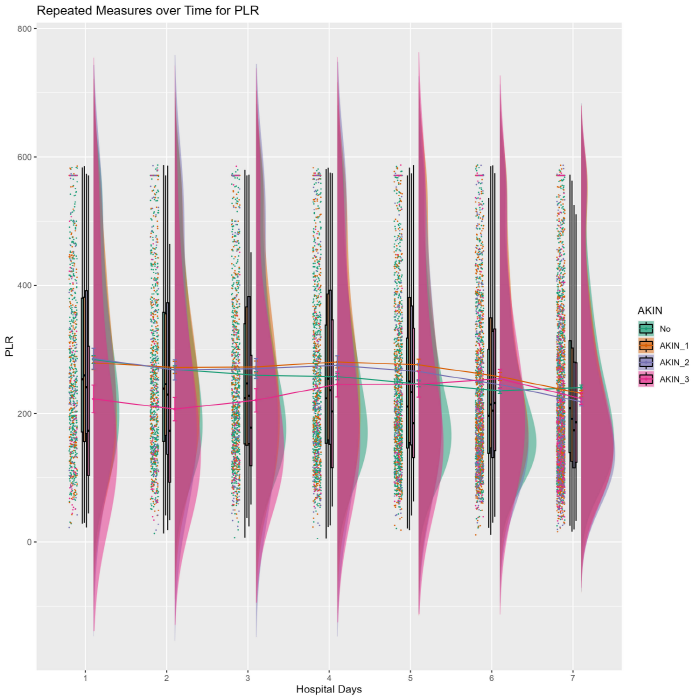


## 21 MLR


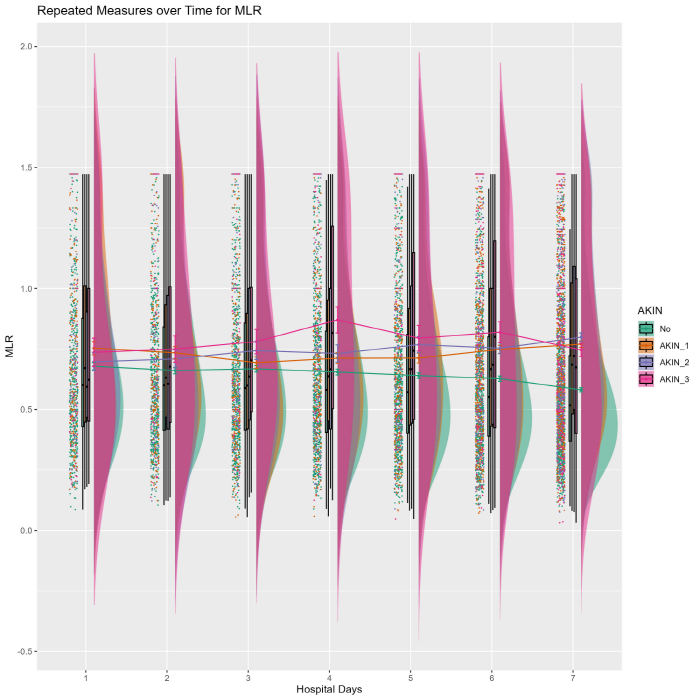


## 22) SII


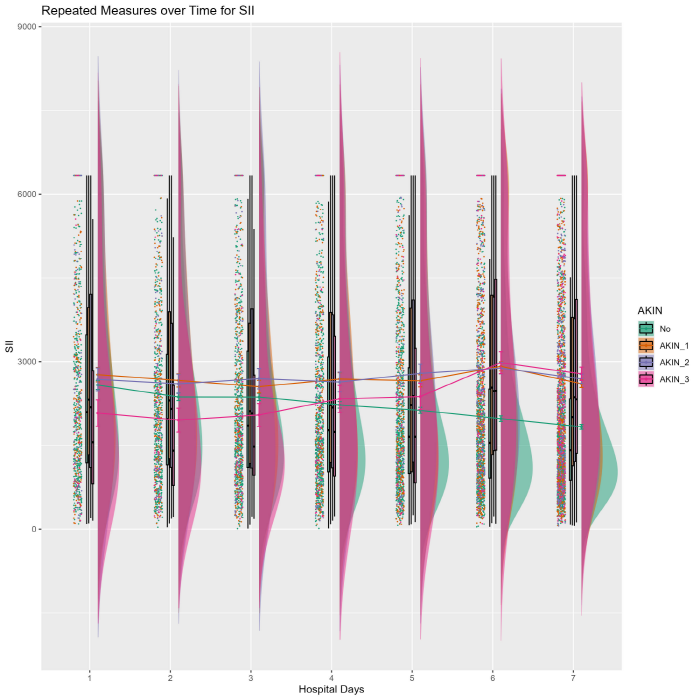


## 23) MPVPR


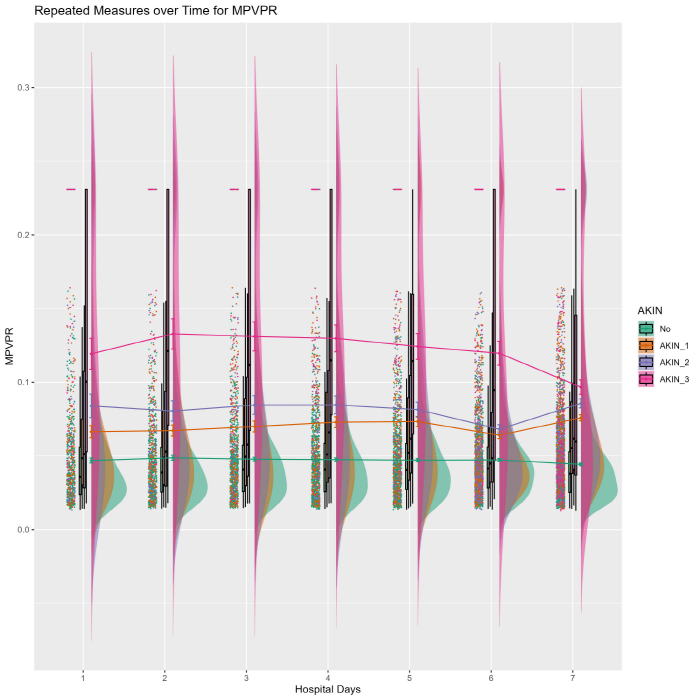


## 24) MPVLR


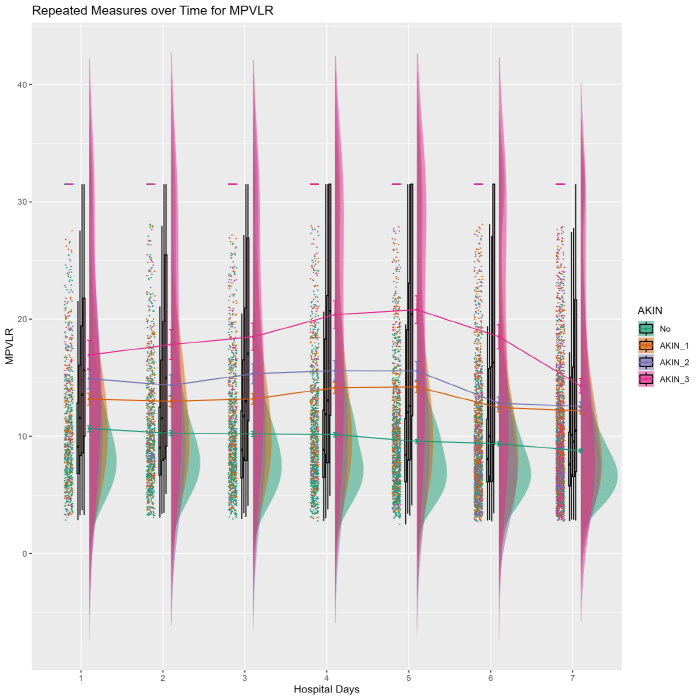


## 25) MPVMR


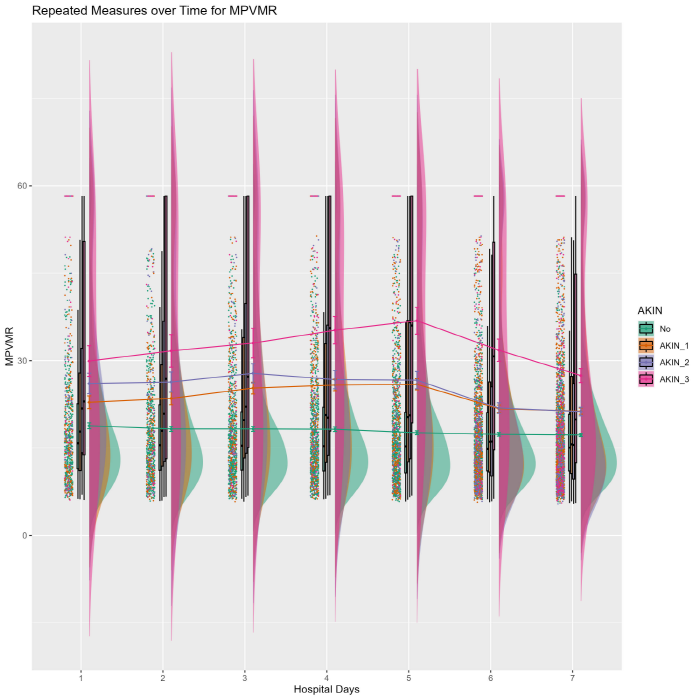


## 26) MPVNR


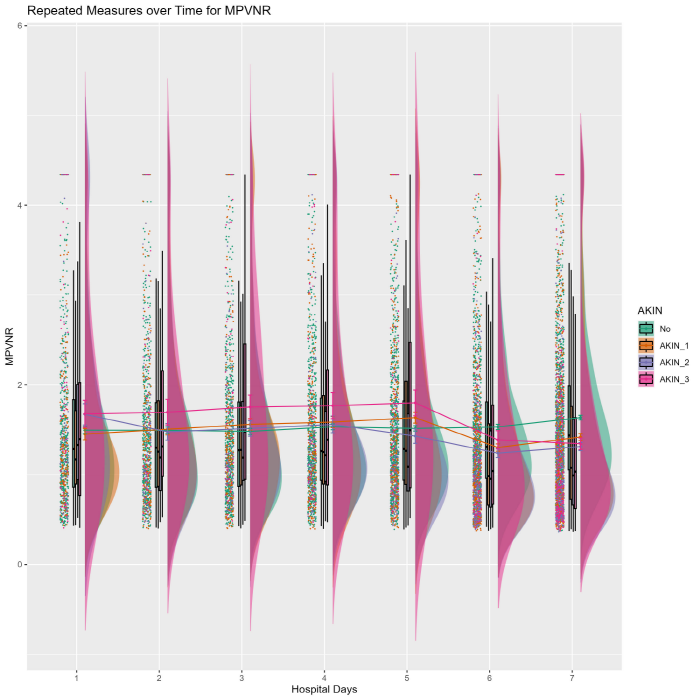

Supplement: Supplementary file 1 — Supplementary Information. [file 41598_2024_51253_MOESM1_ESM.docx]
